# Node-adaptive graph Transformer with structural encoding for accurate and robust lncRNA-disease association prediction

**DOI:** 10.1186/s12864-024-09998-2

**Published:** 2024-01-18

**Authors:** Guanghui Li, Peihao Bai, Cheng Liang, Jiawei Luo

**Affiliations:** 1 School of Information Engineering, East China Jiaotong University, Nanchang, China; 2 School of Information Science and Engineering, Shandong Normal University, Jinan, China; 3 College of Computer Science and Electronic Engineering, Hunan University, Changsha, China

**Keywords:** lncRNA-disease associations, Transformer, Structural deep network embedding, Node-adaptive feature smoothing

## Abstract

**Background:**

Long noncoding RNAs (lncRNAs) are integral to a plethora of critical cellular biological processes, including the regulation of gene expression, cell differentiation, and the development of tumors and cancers. Predicting the relationships between lncRNAs and diseases can contribute to a better understanding of the pathogenic mechanisms of disease and provide strong support for the development of advanced treatment methods.

**Results:**

Therefore, we present an innovative Node-Adaptive Graph Transformer model for predicting unknown LncRNA-Disease Associations, named NAGTLDA. First, we utilize the node-adaptive feature smoothing (NAFS) method to learn the local feature information of nodes and encode the structural information of the fusion similarity network of diseases and lncRNAs using Structural Deep Network Embedding (SDNE). Next, the Transformer module is used to capture potential association information between the network nodes. Finally, we employ a Transformer module with two multi-headed attention layers for learning global-level embedding fusion. Network structure coding is added as the structural inductive bias of the network to compensate for the missing message-passing mechanism in Transformer. NAGTLDA achieved an average AUC of 0.9531 and AUPR of 0.9537 significantly higher than state-of-the-art methods in 5-fold cross validation. We perform case studies on 4 diseases; 55 out of 60 associations between lncRNAs and diseases have been validated in the literatures. The results demonstrate the enormous potential of the graph Transformer structure to incorporate graph structural information for uncovering lncRNA-disease unknown correlations.

**Conclusions:**

Our proposed NAGTLDA model can serve as a highly efficient computational method for predicting biological information associations.

## Background

According to a large number of cell biology experiments, lncRNA are RNA molecule that are not involved in protein coding and exceed approximately 200 nucleotides in length [1–4]. At the beginning of the study, most researchers thought that lncRNAs were just an unimportant product in the transcription process. However, as biological experimental results continue to accumulate, researchers are slowly discovering that lncRNAs are assumed to have very important roles in many important cell biological processes. They are involved in managing the cell cycle, managing embryonic development, the spatial and temporal control of gene expression, determining cell fates [5]. Moreover, researchers in ongoing clinical experiments on human diseases have perceived that lncRNAs are inextricably linked to many human cancers [6, 7] and have a decisive role in human cardiovascular physiological activity and its pathology [8]. Therefore, researchers have regarded lncRNAs as a crucial factor in the study of human diseases and have explored the relationships between diseases and lncRNAs as a new research direction to overcome the barriers of human diseases. Exploring the relationships between diseases and lncRNAs will lead us to deepen our understanding of disease mechanisms [9] and find the causative factors and sources of diseases from the genetic roots. At the same time, understanding the interactions between lncRNAs and diseases will allow us to intervene and regulate the expression of disease-related genes, and find new targets and strategies [10] for the treatment of diseases. Researchers have found that the expression levels of some lncRNAs are very prominent in certain diseases, so lncRNAs can be used as potential biomarkers and play a very important role in the early detection and treatment of diseases. In drug discovery, by exploring the relationship between diseases and lncRNAs, this can help us to investigate new and optimized drugs that are more effective. In addition, human genetic diseases [11] exhibit a close association with lncRNAs. Investigating lncRNAs allows for the elucidation of certain genetic diseases stemming from gene mutations, thereby expediting researchers’ investigations into genetic disorders. However, it requires considerable time to study the linkage in real clinical experiments, requires significant material resources and is challenging to apply on a large scale. Therefore, the design of a novel computational model to compute the association between diseases and lncRNAs is of great importance in advancing the development of bioinformatics. There are some challenges in the actual study, namely: (1) Large datasets exhibit a low percentage of positive samples, resulting in significant sparsity that reduces the model’s ability to predict positive samples effectively. (2) The availability of disease and lncRNA association data is limited, lacking a cohesive fusion of biological association data, and similarity calculations heavily rely on association matrices.

Many methods for calculating lncRNA-disease associations have been developed and their accuracy and reliability have been verified by biological experiments. Thus, to propose better calculation methods, researchers have collected a large quantity of data to create relevant benchmark databases. Gene Reference Into Function (GRIF) [12], DisGeNET [13], and Disease Ontology (DO) [14] are three standard databases related to diseases. RNADisease v4.0 [15], Lnc2Cancer [16] and LncRNADisease [17] are three standard databases related to lncRNA-disease association. These standard databases were also created to break away from the previous way of thinking that one lncRNA corresponds to one disease and to perform global calculations and experiments on the benchmark dataset in the database by the proposed computational method.

Numerous computational techniques for exploring disease-lncRNA interactions have emerged with the continual advancement of diverse technology. We can classify the available computational methods into bioinformatics network-based methods [18] and deep learning-based methods [19].

Bioinformatics network-based models take known associations and their respective similarities to reconstitute heterogeneous networks and use a variety of different messaging mechanisms and random walks for the computation of potential associations on top of the constructed heterogeneity. For example, the KRWRH model [20] utilized the restarted random walks to compute associations between lncRNAs and diseases on top of integrating similarities between diseases, similarities between lncRNAs, and known associations into a new heterogeneous network. The RWRHLD model [21] combined all three of them into a heterogeneous network: observed relationships between lncRNAs and diseases, known associations between crosstalk network between lncRNAs and lncRNAs, and integrating similarity between diseases, based on which links between diseases and lncRNAs are inferred using a restart random walk approach. The IRWRLDA model [22] is a novel algorithm that improves upon traditional random walks by considering both lncRNA similarity and disease similarity for initialization probabilities. It can be used to infer new associations, even when the disease has no known association with any lncRNAs. The SIMCLDA model [23] applied matrix completion and principal component analysis to infer potential associations. The NCPLDA model [24] capitalized on the networks consistency projection to obtain a new computational model for calculating new associations between lncRNAs and diseases. The GrwLDA model [25] generated a global network by combining identified lncRNA-disease interaction information, disease fusion similarity, and lncRNA fusion similarity and utilized this network to explore novel associations between diseases and lncRNAs. The LRWRHLDA model [26] integrated multiple heterogeneous and homogeneous networks to construct a three-layer bioinformatics network using RWR to mine interactions. The LRWHLDA model [27] is designed to excavate the relationships between diseases and lncRNAs with a new idea based on localized random walk that takes full advantage of the topology of the network. The LncRDNetFlow model [28] integrated three interaction networks, disease interaction network, lncRNA interaction network and protein interaction network, to construct a three-layered heterogeneous network to obtain disease and lncRNA feature data. Nevertheless, none of these methods can perform comprehensive learning and fusion of local and global information, nor can they perform deeper network feature learning.

The deep learning-based lncRNA-disease association prediction models have shown significant improvements in performance compared to previous shallow models. The CNNLDA model [29] reorganized multiple sources of similarity and introduced miRNA datasets to enable the neural network model to learn more information. It utilized convolutional neural networks to learn node embeddings and inferred the associations between diseases and lncRNAs. The BiGAN model [30] employed generative adversarial networks for lncRNA-disease interaction calculations. It combined the similarity of lncRNAs and diseases and adopted a bidirectional generative adversarial network to infer their associations. The MCA-Net model [31] utilized embedded learning for multiple feature sources, ensuring that each node has a unique vector representation. It used attention-based convolutional neural networks to excavate direct interactions between lncRNAs and diseases. The ACLDA model [32] constructed a network based on metapaths using lncRNAs, miRNAs, and diseases. It introduced a novel approach that combines CNN and autoencoders for association prediction. The VADLP model [33] constructed multilayer graphs to integrate multiple similarities and employed variance autoencoders and CNN for lncRNA-disease interaction inference. The gGATLDA model [34] utilized attention mechanisms at the graph level. During the graph construction process, each disease-lncRNA pair is extracted to form a subgraph for lncRNA-disease relationship calculation. The MLMKDNN model [35] proposed a deep multi-kernel learning method, which included feature matrix construction, kernel space mapping, and deep neural network fusion. The kernel space mapping technique was applied to transform the feature matrix, enabling effective integration using deep neural networks for fusion. The MLGCNET model [36] employed multilayer graph autoencoder to obtain a representation vector of disease and lncRNA. The MGATE model [37] applied a multi-channel self-attentive encoder to learn latent embeddings of diseases and lncRNAs from multiple angles of the graph. The GANLDA model [38] incorporated multi-source data as initial features. GAT is adopted to get feature information about nodes and their neighbors and finally a multilayer perceptron is leveraged to screen the association. However, when building deep networks in graph neural networks, deep learning tends to cause over-smoothing during the node learning process, resulting in minimal differences between the vector representations of nodes.

A new trend of combining Transformers and graph neural networks to process graph data. This approach combines the parallelizability of Transformers, the advantages of their multi-head attention mechanism, and graph neural network methods to design new neural network models for graph data processing. Microsoft introduced the Graphormer [39], which, for the first time, utilized Transformers for graph-level tasks. It effectively integrated intermediate encoding, spatial encoding, and edge encoding into Transformers, successfully incorporating graph structural information. This integration has shown improved performance in widely used benchmark datasets for graph representation learning. Following this trend, a classic neural network model framework called GraphGPS emerged, which combines graph neural networks and Transformers [40]. It used MLP to learn graph information, feeding it into both the graph neural network and the Transformer for graph representation learning. The fusion of the results obtained from both models leads to highly competitive outcomes.

Although these methods have achieved relatively good results in the task of lncRNA-disease association prediction, they still have limitations and shortcomings as follows: (1) Graph-based methods do not maintain good performance and robustness in the face of sparse large datasets and the problem of over-smoothing of node features can occur [41]. Their learning ability is limited when confronted with complex heterogeneous graphs comprising different nodes and edges [42, 43]. (2) Traditional deep learning-based and bioinformatics network-based approaches do not capture both local and global information, and do not learn the features of nodes by fusing the information encoded in the graph structure. (3) In these existing methods, a simple linear fusion is also used for the fusion of features [23, 24, 26, 38]. The incorporation of adaptive and efficient fusion approach holds the potential for significant improvements in model performance and robustness.

Based on the aforementioned limitations of the existing methods and the inherent advantages of the Transformer model, we propose an innovative lncRNA-disease association prediction model named NAGTLDA. First, we construct a heterogeneous network by utilizing observed associations and compute the integrated similarity of diseases and lncRNAs to create their respective integrated similarity networks. Next, we employ node-adaptive feature smoothing (NAFS) [44] to perform local-level node embedding on the heterogeneous network and integrated similarity networks. Simultaneously, we utilize Structural Deep Network Embedding (SDNE) [45] to encode the structural information of the integrated similarity networks. Furthermore, we utilize the Transformer model for global-level embedding learning, allowing it to leverage its inherent global perspective to unearth potential association information. Finally, we employ the Transformer model to perform global-level fusion of all learned embeddings and incorporate the structural inductive bias of the network. This fusion approach effectively and significantly enhances the utilization of all captured information, thereby greatly improving the performance of inferring the associations between diseases and lncRNAs. Our proposed model outperforms these models that exist now in terms of performance and scalability.

In summary, our research makes the following key contributions:

- We employ the NAFS method for feature embedding learning without the need for explicit training, and we utilize SDNE to encode the network structure.
- We employ both local-level and global-level approaches for feature embedding, enabling the model to effectively uncover potential association information.
- To improve the Transformer model for learning graph node information, we learn the network’s structural information as an inductive bias.
- We propose a Transformer fusion mechanism, which introduces the Transformer model for node embedding and fusion of multiple features and topology information, enriching the representation of lncRNAs and diseases.

## Methods

### Known human lncRNA-disease associations

In our experiment, we used a benchmark dataset to assess the effectiveness of our model. This dataset was obtained from previous research by Fu et al. [46] on lncRNA-disease association prediction, which includes 240 lncRNAs, 412 diseases, and 2697 experimentally validated lncRNA-disease interactions from the Lnc2Cancer [16], LncRNADisease [17], and GeneRIF [47] databases. We denoted the quantity of diseases and lncRNAs as ${N}_{l}$ and ${N}_{d}$, respectively. We constructed an adjacency matrix *A* based on the observed interactions between lncRNAs and diseases, and $A\in {R}^{{N}_{l}\times {N}_{d}}$, where $A(l(i), d(j))=1$ if there exists an identified relationship between lncRNA $l(i)$ and disease $d(j)$; otherwise $A(l(i), d(j))=0$.

### LncRNA functional similarity

There are multiple methods for expressing the similarity between lncRNAs, and one common method is based on their association with related diseases. By comparing the similarity of different lncRNAs with their associated diseases, their functional similarity can be assessed. In this experiment, we adopted the lncRNA functional similarity calculation method proposed by Chen et al. [48], which assumes that there are two lncRNAs ${l}_{1}$ and ${l}_{2}$, respectively, ${l}_{1}$ is linked to disease category $D(i)=\left\{{d}_{i1}, {d}_{i2}, {d}_{i3},\cdots , {d}_{in}\right\}$, and ${l}_{2}$ is linked to disease category $D(j)=\left\{{d}_{j1}, {d}_{j2}, {d}_{j3},\cdots , {d}_{jm}\right\}$. The formula for calculating the similarity score between disease ${d}_{k}\in {\text{D}}({\text{i}})$ and disease category $D(j)$ provided here is:

1 $$S({d_k},D(j)) = max(D{S_{d \in D(j)}}({d_k},d))$$

where $DS({d}_{k}, d)$ represents the semantic similarity between diseases ${d}_{k}$ and *d*. Based on the semantic similarity between the diseases and the associations between the lncRNAs and disease category, the formula for calculating the functional similarity of lncRNAs is as follows:

2 $$LF({l_i},{l_j}) = \frac{{\sum\nolimits_{d \in D(i)} {S(d,D(j)} + \sum\nolimits_{d \in D(j)} {S(d,D(i))} }}{n + m}$$

where *n* and *m* denote the quantity of diseases in disease category $D(i)$ and category $D(j)$, which can be represented as $|D(i)|=n,$ |$D(j)|=m$, respectively.

### Disease semantic similarity

To compute the semantic similarity between diseases, their Medical Subject Headings (MeSH) descriptors can be used [49], and they can be denoted as a Directed Acyclic Graph (DAG) [50]. Specifically, the hierarchical relationship of a disease can be represented as $DAG({d}_{i})=(T({d}_{i}), E({d}_{i}))$, where $T({d}_{i})$ represents ${d}_{i}$ and all its ancestor nodes, and $E({d}_{i})$ is a set of edges from ancestral nodes to descendant nodes. Computing disease semantic similarity can be divided into three steps. For the first stage, for any disease ${d}_{j}$ in $DAG({d}_{i})$, its contribution towards the semantic similarity of disease ${d}_{i}$ can be computed using the following formula:

3 $${S_{d_i}}({d_j}) =\left\{\begin{array}{cc}1&if \, {d_j} = {d_i}\\\text{max}\left\{\gamma\ast{S_{d_i}}\left(d^\prime_j\right)|d^\prime_j\,\epsilon\,children\,of\,d_i\right\}&if \, {d_j} \neq {d_i}\end{array}\right.$$

where parameter $\gamma$ represents a hyperparameter set to 0.5 in the formula for disease semantic contribution. The second stage is to compute the total semantic value of the disease, which is computed using the following formula for ${DV}_{{d}_{i}}$:

4 $$D{V_{d_i}} = \sum\nolimits_{d \in T({d_i})} {{S_{d_i}}(d)}$$

The third stage is to compute the semantic similarity between diseases ${d}_{i}$ and ${d}_{j}$ using the following formula:

5 $$DS({d_i},{d_j}) = \frac{{\sum\nolimits_{d \in T({d_i}) \cap T({d_j})} {({S_{d_i}}(d) + {S_{d_j}}(d))} }}{{D{V_{d_i}} + D{V_{d_j}}}}$$

### Gaussian interaction profile (GIP) kernel similarity for lncRNAs and diseases

Gaussian kernel similarity is a common similarity measurement method that can map data to a multidimensional space and compute the similarity between data points. The calculated lncRNA functional similarity and disease semantic similarity are both relatively sparse, so it is necessary to introduce other similarities to compensate for this deficiency. Therefore, we decided to introduce GIP similarity, which can make the similarity between data nodes more obvious and facilitate the prediction of associations between nodes. The calculation formulas for GIP kernel similarity $LK({l}_{i}, { l}_{i})$ between lncRNA ${l}_{i}$ and ${l}_{j}$ and DK (${d}_{i}$, ${d}_{j}$) between disease ${d}_{i}$ and ${d}_{j}$ are as follows:

6 $$LK({l_i},{l_j}) = exp( - {r_l} {\text{P}}IP({l_i}) - IP({l_j}){\text{P}^2})$$

7 $$DK({d_i},{d_j}) = exp( - {r_d}\text{P} IP({d_i}) - IP({d_j}){\text{P}^2})$$

where comparable to reference [51], $IP({l}_{i})$ and $IP({ l}_{j})$ represent the *i*-row and *j*-row corresponding to the lncRNA in the known lncRNA-disease interaction matrix *A*,$IP({d}_{i})$ and $IP({d}_{i})$ represent the *i*-column and *j*-column corresponding to the disease in the known lncRNA-disease interaction matrix* A*. ${r}_{l}$ and ${r}_{d}$ are the kernel bandwidth control parameters and are defined by the following formula:

8 $${r_l} = r_l^\prime/(\frac{1}{{N_l}}{\sum\nolimits_{i = 1}^{N_l} {\text{P} IP({l_i})}\text{P}^2})$$

9 $${r_d} = r_d^\prime/(\frac{1}{{N_d}}{\sum\nolimits_{i = 1}^{N_d} {\text{P} IP({d_i})}\text{P}^2})$$

### Integrated similarity networks for lncRNAs and diseases

Previously, we introduced GIP kernel similarity to compensate for the sparsity of lncRNA functional similarity and disease semantic similarity. Based on these similarities, we calculate the integrated similarity matrix between diseases and lncRNAs using the following formula:

10 $$IL({l_i},{l_j}) = \left\{ {\begin{array}{*{20}{c}} {LF({l_i},{l_j})}&{if \, {l_i} \, and \, {l_j} \, have \, functional \, similarity} \\ {LK({l_i},{l_j})}&{otherwise} \end{array}} \right.$$

11 $$ID({l_i},{l_j}) = \left\{ {\begin{array}{*{20}{c}} {DS({d_i},{d_j})}&{if \, {d_i} \, and \, {d_j} \, have \, semantic \, similarity} \\ {DK({d_i},{d_j})}&{otherwise} \end{array}} \right.$$

where $IL({l}_{i}, { l}_{j} )$ represents the integrated similarity matrix between lncRNAs, and $ID({d}_{i}, {d}_{j})$ represents the similarity matrix between diseases. To better utilize the integrated similarity matrices of lncRNAs and diseases, we use them to obtain their corresponding integrated similarity networks. We set two thresholds $\alpha$ and $\beta$ to calculate the similarity network, and their formulas are expressed as follows:

12 $${I_{net}}({l_i},{l_j}) = \left\{ {\begin{array}{*{20}{c}} 1&{if \, IL({l_i},{l_j}) \geqslant \alpha } \\ 0&{otherwise} \end{array}} \right.$$

13 $${D_{net}}({d_i},{d_j}) = \left\{ {\begin{array}{*{20}{c}} 1&{if \, ID({d_i},{d_j}) \geqslant \beta } \\ 0&{otherwise} \end{array}} \right.$$

where ${I}_{net}$ represents the network obtained from the integrated similarity matrix of lncRNAs. If the similarity value between ${l}_{i}$ and ${l}_{j}$ is not less than or equal to threshold $\alpha$, then ${I}_{net}({l}_{i}, { l}_{j})$ = 1. Otherwise, ${I}_{net}({l}_{i}, { l}_{j})$ = 0. ${D}_{net}$ denotes the network obtained from the integrated similarity matrix of diseases. If the similarity value between ${d}_{i}$ and ${d}_{j}$ is not less than or equal to threshold $\beta$, then ${D}_{net}({d}_{i}, {d}_{j})$=1. Otherwise, ${D}_{net}({d}_{i}, {d}_{j})$=0.

### LncRNA-disease heterogeneous network

We constructed a lncRNA-disease heterogeneous network that includes the lncRNA similarity matrix, disease similarity matrix, and the known lncRNA-disease association matrix* A*:

14 $${G_{net}} = \left[ {\begin{array}{*{20}{c}} {IL}&A \\ {A^T}&{ID} \end{array}} \right] \in {R^{({N_l} + {N_d}) \times ({N_l} + {N_d})}}$$

where ${A}^{T}$ represents the transpose of the lncRNA-disease interaction matrix.

### NAGTLDA

**Fig. 1:**
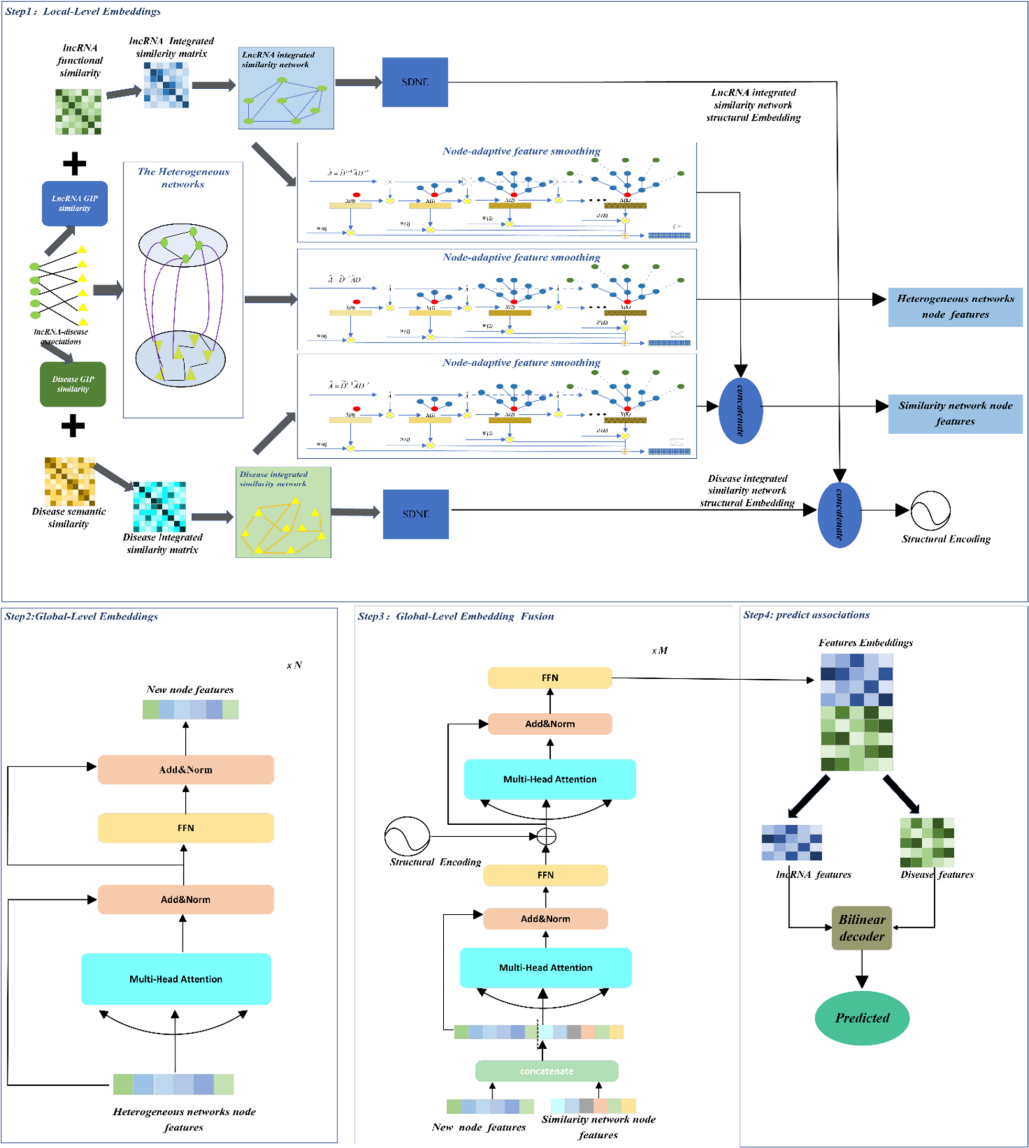
The NAGTLDA workflow. Step1: Construct the integrated similarity network, extract the local features of the heterogeneous network and the integrated similarity network adopting NAFS, and encode the structural information of the integrated similarity network applying SDNE. Step2: Learn global information of heterogeneous network nodes by Transformer architecture. Step3: Adaptively fusing local information of nodes, global information and structural coding of the network by Transformer architecture. Step4: Predict associations using bilinear encoder

This section provides a detailed introduction to our proposed model, NAGTLDA, which accurately excavates the lncRNA-disease associations. The NAGTLDA process is shown in Fig. 1, which depicts the workflow and the sequence of steps involved in the NAGTLDA framework. The model framework comprises the following parts: (1) using NAFS to learn local-level node feature embedding, (2) using SDNE to encode the structure of networks, (3) using a Transformer model with a multi-head attention layer to learn global-level node feature embedding, (4) using a Transformer model with two multi-head attention layers to learn embedding fusion at the global-level, (5) predicting the association score between diseases and lncRNAs.

### Local-level node feature embedding (node-adaptive feature smoothing)

In recent years, GCN [52] has become very popular in graph neural networks (GNNs). This is because GCN can learn the features of all nodes in a graph based on both node features and graph structure. Using GCN to aggregate multi-order neighbour information in large graph networks leads to over-smoothing problems and requires a high computational cost and large memory consumption. To address this issue, Zhang et al. [44] proposed a model called NAFS, which aggregates and updates the features of nodes in a graph. Compared with GCN, NAFS not only solves the limitations of GCN but also significantly simplifies the model training intricacy and mitigates the occurrence of gradient vanishing and gradient explosion during backpropagation without the need for additional training.

Since our model uses NAFS for node feature embedding for all three graphs (${I}_{net},{D}_{net } \,and\, {G}_{net}$), we use ${G}_{net}$ as an example for illustration. The abbreviation for ${G}_{net}$ is *G*. We denote the quantity of nodes in *G* as *n* and the quantity of edges as *m*. Computing of NAFS consists of four steps. The initial step entails computing the over-smoothing distance, and the calculation is performed in the following manner:

15 $${D_i}(k) = Dis({[{\hat G^K}X]_i},{[{\hat G^\infty }X]_i})$$

where ${[{\widehat{G}}^{k}X]}_{i}$ represents the *i*-th row in the matrix, which indicates the smoothed node representation of the *i*th node. *Dis*(•) represents a distance formula, which can be implemented using the Euclidean distance formula. $\widehat{G}={\widetilde{D}}^{r-1}\widetilde{G}{\widetilde{D}}^{-r}$,$\widetilde{D}$ denotes the degree matrix of graph. *r* is a hyperparameter in the model. $\widetilde{G}$ represents the adjacency matrix of the undirected graph with self-loops added. The calculation formula for ${\widehat{G}}^{\infty }$ is as follows:

16 $$\hat G_{i,j}^\infty = \frac{{{{({d_i} + 1)}^r}{{({d_j} + 1)}^{1 - r}}}}{2m + n}$$

where ${d}_{i}$ represents the degree of node *i*. The smoothing weight calculated in the second step is computed as follows:

17 $${\omega_i}(k) = \mathop e\nolimits^{{D_i}(k)} /\sum\nolimits_{l = 0}^k {{e^{{D_i}(l)}}}$$

where *K* represents the maximum number of smoothing steps. The third step is to calculate the smoothing weight matrix, which is computed as follows:

18 $$W(k) = Diag(\varphi (k)), \, \varphi (k)[i] = {\omega_i}(k), \, \forall 1 \leq i \leq n$$

where $\varphi (k)\in {R}^{n}$ and $Diag(\cdot )$ represents a diagonal matrix. We denote the initial input feature representation as ${X}^{(0)}$. After $l$ rounds of smoothing, the node feature matrix ${X}^{(l)}=\widehat{G}{X}^{(l-1)}$ contains the feature of the previous round of smoothing. After* K* rounds of maximum smoothing, ${X}^{(k)}$ will contain more information, and we can obtain a collection of feature matrices $\left\{{X}^{(0)}, \,{X}^{(1)}, \,{X}^{(2)}, \,\cdots , \,{X}^{(k)}\right\}$. Finally, the formula for smoothing feature $\widehat{X}$ is as follows:

19 $$\hat X = \sum\nolimits_{l = 0}^K {W(l){X^{(l)}}}$$

The definition of ${X}^{(0)}$ is as follows:

20 $${X^{(0)}} = \left[ {\begin{array}{*{20}{c}} 0&A \\ {A^T}&0 \end{array}} \right]$$

In GCN, a symmetric normalized adjacency matrix $\widehat{G}={\widetilde{D}}^{r-1}\widetilde{G}{\widetilde{D}}^{-r}$ is used. Setting *r* = 0.5 yields the symmetric normalized adjacency matrix ${\widetilde{D}}^{-1/2}\widetilde{G}{\widetilde{D}}^{-1/2}$ [52] as the feature extractor. However, in NAFS, $\left\{{r}_{1}, \,{r}_{2}, \,{r}_{3}, \,\cdots , \,{r}_{U}\right\}$ results in a more diverse set of feature embeddings. The value of *r* controls the normalization weight of each edge, so different *r* values lead to distinct node feature embeddings for the same graph. We obtain a set of smoothed features $\left\{\widehat X^{(0)},\widehat X^{(1)},\widehat X^{(2)},\cdots,\widehat X^{(U)}\right\}$ based on this set of different *r* values, and we combine different smoothed features into ${\widehat{Z}}_{G}=({\widehat{X}}^{(0)}\otimes {\widehat{X}}^{(1)}\cdots \otimes {\widehat{X}}^{(U)})\in {{\varvec{R}}}^{({N}_{l}+{N}_{d})\times ({N}_{l}+{N}_{d})}$. Here, $\otimes$ represents a type of combination method, which can be replaced with the max function, concatenation, and mean function.

First, we input the heterogeneous network ${G_{net}} \in {R^{({N_l} + {N_d}) \times ({N_l} + {N_d})}}$ and the initial features ${X^{(0)}} \in {R^{({N_l} + {N_d}) \times ({N_l} + {N_d})}}$ of the network nodes, which consists of nodes corresponding to lncRNAs and disease entities. We will compute a smoothing weight matrix $W(k)$ for each *k-step* according to Eq. (18), then we use a list $\left\{{r}_{1}, \,{r}_{2}, \,{r}_{3},\,\cdots,\,{r}_{U}\right\}$. For each *r*-value in the list, we derive a new feature node embedding representation of the network structure from Eq. (19), denoted as ${\hat X^{(u)}} \in {R^{({N_l} + {N_d}) \times ({N_l} + {N_d})}}$. The feature embeddings obtained from all the *r*-value are fused to obtain the final feature embedding ${\hat Z_G} \in {R^{({N_l} + {N_d}) \times ({N_l} + {N_d})}}$. The final NAFS is expressed as follows:

21 $$NAFS = ({\hat X^{(0)}} \otimes {\hat X^{(1)}} \otimes \cdots \otimes {\hat X^{(U)}})$$

where *U* denotes the length of the *r*-list and $\otimes$ represents the fusion mode of the features (Mean).

Similarly, we use NAFS to process and obtain the corresponding lncRNA-integrated similarity network node features ${\hat Z_L} \in {\varvec{R}^{{N_l} \times{N_l}}}$ and disease-integrated similarity network node features ${\widehat{Z}}_{D}\in {{\varvec{R}}}^{{N}_{d}\times {N}_{d}}$. We perform the node features in ${\widehat{Z}}_{L}$ affine, converting ${\widehat{Z}}_{L}$ and ${\widehat{Z}}_{D}$ to the same dimension:

22 $$\hat Z_L^\prime(i) = {W_{LD}}{\hat Z_L}(i) + {b_{LD}}$$

where ${W}_{LD}\in {{\varvec{R}}}^{{N}_{d}\times {N}_{l}}$ and ${b}_{LD}\in {{\varvec{R}}}^{{N}_{d}}$ are trainable parameters. We splice ${\widehat{Z}}_{L}^\prime$ and ${\widehat{Z}}_{D}$ to form a new node feature $${\widehat{Z}}_{LD}=\left[\begin{array}{c}{\widehat{Z}}_{L}^\prime\\ {\widehat{ Z}}_{D}\end{array}\right]\in {R}^{({N}_{l}+{N}_{d})\times {N}_{d}}$$.

### Network structure encoding

We learn the structural encoding of the network as the structural inductive bias and transfer it to the downstream Transformer module for processing. Here, we encode the network structure using the SDNE approach provided by Wang et al. [45] to conduct additional research on the information in the network.

In the model we encode the structure of the network with ${I}_{net}\mathrm{ \,and \,}{D}_{net}$. Here we use ${I}_{net}$ as an example to illustrate the process of SDNE. SDNE is composed of a decoder part and an encoder, where the decoder maps the input network with multiple nonlinear functions and the decoder applies multiple nonlinear functions to reconstruct the network. In ${I}_{net}=(V, E)$, the adjacency matrix of the network is denoted by $M$, $V$ denotes the collection of lncRNA nodes within the network, where $|V|={N}_{l}$. Then, the mapping and reconstruction of the network is performed as follows:

23 $$\begin{array}{ll} y_i^{(1)} = \sigma (W_l^{(1)}{M_i} + {b^{(1)}}) \hfill \\ y_i^{(k)} = \sigma (W_l^{(k)}y_i^{(k - 1)} + {b^{(k)}}), \, k = 2, L ,K \hfill \\ \end{array}$$

where ${M}_{i}$ denotes the initial feature of the *i*th lncRNA in the network, $\upsigma \left(\cdot \right)$ denotes the activation function, ${W}_{l}^{\left(1\right)}\in {{\varvec{R}}}^{{n}_{1}\times {N}_{l}}, {b}^{(1)}\in {{\varvec{R}}}^{{n}_{1}}$,${W}_{l}^{(k)}\in {{\varvec{R}}}^{{n}_{k}\times {n}_{k-1}}$ and ${b}^{(k)}\in {{\varvec{R}}}^{{n}_{k}}$ are the trainable parameters, and *K* is the number of layers of the decoder and encoder hidden layers. When ${y}_{i}^{(k)}$ is obtained, the encoder will be reused to map to obtain the output ${\widehat{M}}_{i}$. To make SDNE capture a more accurate network structure, second-order similarity and first-order similarity are used here to construct the loss function of SDNE so that the error between the reconstructed network and the original network is smaller, and the SDNE loss function ${L}_{sdne}$ is calculated as follows:

24 $$\begin{gathered} {L_{2nd}} = \sum\nolimits_{i = 1}^{N_l} {\text{P} ({{\hat M}_i} - {M_i}) \text{e}\;{b_i}\text{P} }_2^2 \\ \, = \text{P} (\hat M - M)\text{e}\;B\text{P}_F^2 \\ \end{gathered}.$$

25 $$\begin{gathered} {L_{1st}} = \sum\nolimits_{i,j = 1}^{N_l} {M(i,j)\text{P} y_i^{(k)} - y_j^{(k)}\text{P}_2^2} \\ = M(i,j)\text{P} {y_i} - {y_j}\text{P}_2^2 \\ \end{gathered}$$

26 $${L_{sdne}} = {L_{2nd}} + \alpha {L_{1st}} + {L_{reg}}$$

Here, ⊙ represents the Hadamard product. ${b}_{i}={\{{b}_{i,j}\}}_{j=1}^{{N}_{l}}$, if $M(i,j)$=0, ${b}_{i,j}$=1; otherwise, ${b}_{i,j}=\beta >1$. $M$ represents the adjacency matrix of the network, $M(i, j)$ represents the value of the ith row and jth column of the association matrix, and $\alpha$ is the hyperparameter. ${L}_{reg}$ is a regularization term proposed to avoid overfitting, which is calculated as follows:

27 $${L_{reg}} = \frac{1}{2}\sum\nolimits_{k = 1}^K {(\text{P} W_l^{(k)}\text{P}_F^2 + \text{P} {{\hat W}_l}^{(k)}\text{P}_F^2)}$$

We input a network $G = (V,E)$, where *V* denotes the set of nodes and *E* denotes the set of edges. Encode the network structure following the formulation in Eq. (23). Subsequently, decode the network structure by passing it through a decoding module, utilizing Eq. (26). Employ Eq. (24) for the first-order loss function, Eq. (25) for the second-order loss function, and Eq. (27) for the regularization function to compute the loss of the reconstructed network structure. This comprehensive approach aims to enhance the accuracy of the encoded network structure. Finally, output the result $y_i^{(k)}$ obtained from the encoder. ${{I}_{net}\mathrm{ \,and \,}D}_{net}$ denote lncRNA-integrated similarity network and disease-integrated similarity network. The final expression of the SDNE is as follows:

28 $$\hat M = \{ SDNE_L^K({M_i})\}_{i = 1}^{N_l}$$

29 $$\hat D = \{ SDNE_D^K({D_j})\}_{j = 1}^{N_d}$$

where $\widehat{M}\in {{\varvec{R}}}^{{N}_{l}\times {n}_{p} } \,{\text{and}} \,\widehat{D}\in {{\varvec{R}}}^{{N}_{d}\times {n}_{p}}$, ${n}_{p}=K/2$, and *K* denotes the number of hidden layers in the decoder and encoder. We combine $\widehat{M}$ and $\widehat{D}$ into a new network structure coding $$SF=\left[\begin{array}{c}\widehat{M}\\ \widehat{D}\end{array}\right]\in {{\varvec{R}}}^{({N}_{l}+{N}_{d})\times {n}_{p}}$$.

### Global-level embedding

In our model, we account for the limitations of the information contained in the local-level nodes. Therefore, we introduce a Transformer [53] module to learn global-level node features and deeply explore the unknown associations between diseases and lncRNAs from a global perspective. The Transformer is utilized in the domain of graph neural networks and has significant implications for the future development of graph neural networks. In NAGTLDA, we only need the Transformer encoder to learn the feature embedding of the global-level nodes.

We take the node features ${\widehat{Z}}_{G}$ of the heterogeneous network as input to the Transformer, which is first processed through the multi-head attention layer as follows:

30 $${Q_i} = {\hat Z_G}W_i^q, \, {K_i} = {\hat Z_G}W_i^k, \, {V_i} = {\hat Z_G}W_i^v$$

31 $${H_i} = (\frac{{\exp ({Q_i}K_i^T/\sqrt d )}}{{\sum\nolimits_{j = 1}^{{n_{head}}} {\exp (({Q_j}K_j^T)/\sqrt d )} }}){V_i}$$

where ${W}_{i}^{q}$*, *${W}_{i}^{k}$*, *${W}_{i}^{v}\in {R}^{({N}_{l}+{N}_{d})\times (({N}_{l}+{N}_{d})/{n}_{head})}$ are the parameters to be trained in the model and ${n}_{head}$ represents the quantity of multi-head attention heads. We obtain a set ${{H}_{i}=\{H}_{1}, {H}_{2}, \cdots , {H}_{{n}_{head}}\}$, and finally, we obtain the output *H* from the multi-head attention:

32 $$H = ({H_1} \oplus {H_2} \oplus \cdots \oplus {H_{{n_{head}}}}) \cdot {W^H}$$

where, ${W}^{H}\in {R}^{({N}_{l}+{N}_{d})\times {n}_{h}}$ is the training parameter and $\oplus$ represents the splicing operation. Then we feedforward propagate the output of the multi-head attention, and the feedforward network is defined as follows:

33 $$FF{N_i}(T) = \sigma (T{W^{Fi}} + {b^{Fi}})$$

where $\sigma (\cdot )$ represents a nonlinear activation function (LeakyReLU) and *i* denotes the quantity of hidden layers in the feedforward network. Here, given the initial input $H$, we can proceed to obtain the output *X* of the feedforward network:

34 $${X_m} = LeakyRelu(H{W^{F1}} + {b^{F1}})$$

35 $$X = LeakyRelu({X_m}{W^{F2}} + {b^{F2}})$$

where ${W}^{F1}\in {R}^{{n}_{h}\times {n}_{d}}$*, *${W}^{F2}\in {R}^{{n}_{d\times }{n}_{h}}$*, *${b}^{F1}\in {R}^{{n}_{d}}\mathrm{\, and \,}{b}^{F2}\in {R}^{{n}_{h}}$ are the training parameters.

### Global-level embedding fusion

We have acquired local-level and global-level embeddings, and as it would be inefficient to combine these various embeddings using straightforward splicing or summing operations to produce the desired result, we continue to employ Transformer’s decoder to carry out global-level node embedding fusion representation. Transformer does not employ the graph information transfer mechanism for graph computation; as a result, the structural inductive bias of the network is introduced to Transformer to compensate for the missing information transfer mechanism, resulting in excellent results for the model. Here, we employ two multi-headed attention layers, the first of which handles node embedding and the second of which incorporates structural inductive bias of the network for developing the final node embedding representation learning.

First, we use the first multi-head attention layer to process the concatenation of the global-level embedding *X* and the local-level embedding ${\widehat{Z}}_{LD}$. By applying the multi-head attention Eqs. (30), (31), and (32) along with the feedforward network Eq. (33) we obtain a new node embedding ${X}^{F}\in {R}^{({N}_{l}+{N}_{d})\times {n}_{h}^{\mathrm{^\prime}}}$.

Then, we use the second layer of multi-head attention to address the structural induction bias of the network. After concatenating the structural induction bias SF and node embedding ${X}^{F}$, we similarly utilize Eqs. (30), (31), (32) for multi-head attention and Eq. (33) for the feedforward network to obtain a new representation of the node embedding ${X}^{S}$.

We utilized the rich information of the heterogeneous network and the topological structure of integrated similarities networks for lncRNAs and diseases to perform node feature embedding learning at both local-level and global level. Simultaneously, we learned the structural information of the network. Finally, we fuse them using the Transformer structure to obtain the final node embedding representation ${X}^{S}\in {R}^{{(N}_{l}+{N}_{d})\times f}$.

### Predicting the association score between lncRNAs and diseases

We expressed the final node embedding expression as $${X}^{S}=\left[\begin{array}{c}{X}_{L}^{S}\\ {X}_{D}^{S}\end{array}\right]$$, where ${X}_{L}^{S}\in {R}^{{N}_{l}\times f}$ indicates the ultimate node feature embedding of lncRNAs and ${X}_{D}^{S}\in {R}^{{N}_{d}\times f}$ indicates the ultimate node feature embedding of diseases. The reconstruction of the lncRNA-disease interaction matrix $\widehat{A}$ was performed using a bilinear decoder. The bilinear decoder formula is defined as follows:

36 $$\hat A = sigmoid(X_L^S{W^B}X_D^S)$$

where ${W}^{B}$ represents the trainable parameter matrix. We can consider the lncRNA-disease link prediction task as a simple binary classification problem, so binary cross-entropy loss is selected as the loss function for association prediction, which is calculated as follows:

37 $${L_{l\_p}} = - \sum\nolimits_{(i,j) \in {I^+ } \cup {I^- }} {\{ A(i,j)\ln \hat A(i,j) + [1 - \hat A(i,j)]\ln [1 - \hat A(i,j)]\} }$$

where (*i, j*) denotes the lncRNA and disease pairs, and the sets of data that are negative and positive data are represented by ${I}^{-}$ and ${I}^{+}$, respectively. Our model’s overall loss function can be described as follows:

38 $${L_m} = {L_{l\_p}} + L_{sdne}^1 + L_{sdne}^2$$

where ${L}_{l\_p}$ stands for the loss function of the reconstructed association matrix, whereas ${L}_{sdne}^{1}$ and ${L}_{sdne}^{2}$ reflect, the loss functions represented by the structures of the disease-integrated similarity and lncRNA-integrated similarity networks, respectively. In the overall optimization of our model, we added the Adam optimizer [54]. To achieve an equal distribution of negative and positive samples during the training phase of our model, an equivalent quantity of negative data is randomly chosen to enter the training. The training process of NAGTLDA is shown in Algorithm 1.

**Figure:**
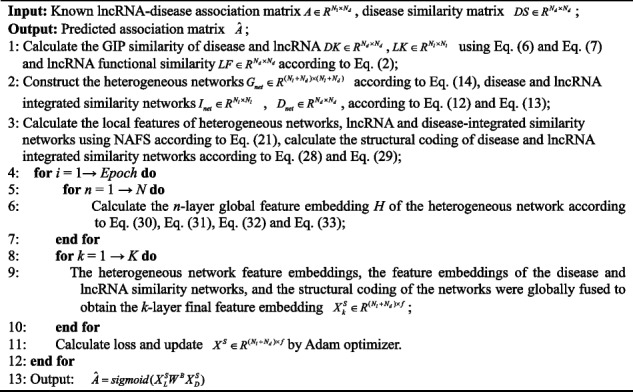
**Algorithm 1.** Algorithm of our proposed method

## Results

### Experimental setting

**Fig. 2:**
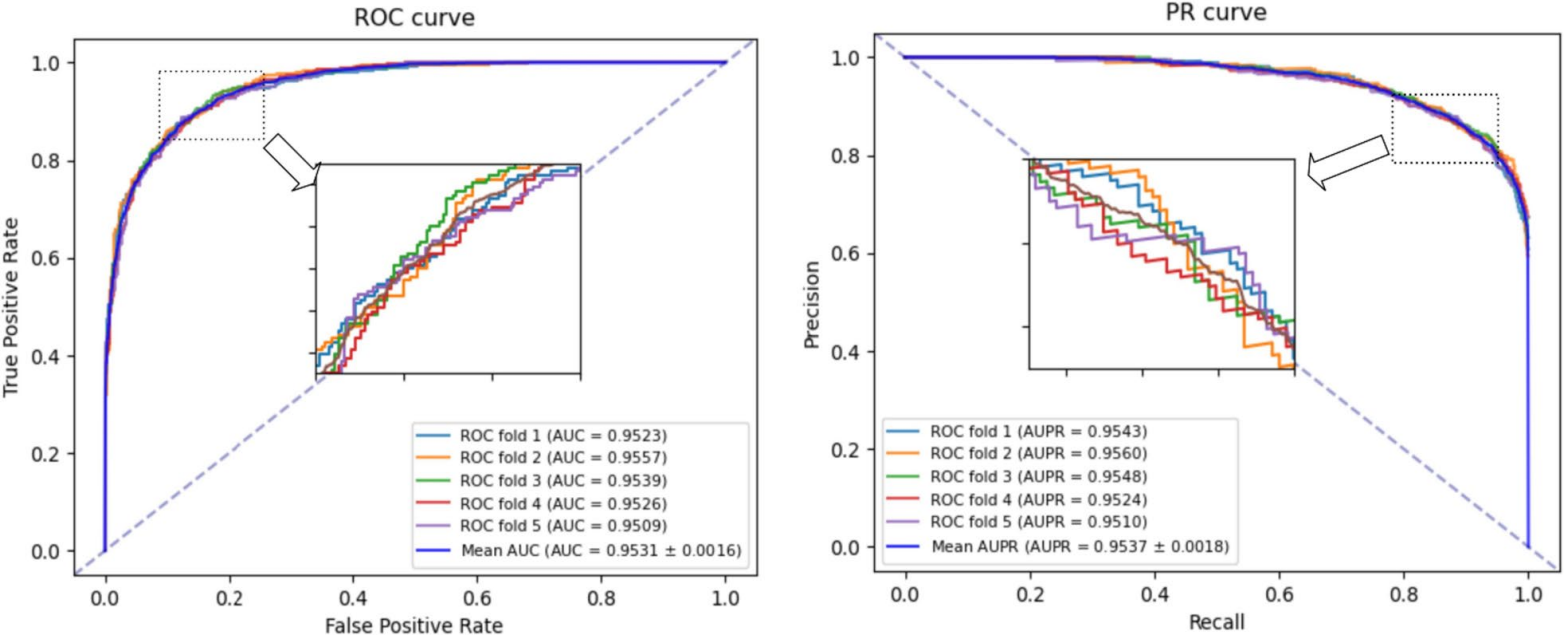
ROC curves and PR curves of NAGTLDA in 5-CV

During our experimental process, we employed 5-fold cross-validation (5-CV) to test the performance of our proposed model. We partitioned the disease-lncRNA pairs into five equal subsets, employing a four-to-one ratio for training and testing, which facilitated five cross-validation iterations. In each round, we removed all known associations from the test set and evaluated the performance of the trained model on the test samples. For selecting performance evaluation metrics, we adopted AUPR (area under precision-recall curve) and AUC (area under the receiver operating characteristic curve) as the major markers. Additionally, we considered five auxiliary reference metrics: recall, accuracy (ACC), F1-score, precision (Prec.), and specificity (Spec.). After conducting our 5-CV experiment, detailed results are presented in Table 1. Our model achieved an average accuracy of 0.8785 and average recall of 0.9088 on the experimental dataset. The average specificity and precision reached 0.8483 and 0.8578, respectively, while the average F1-score reached 0.882. In particular, the AUC and AUPR for our model are shown in Fig. 2. The average AUC and AUPR were 0.9531 and 0.9537, respectively. The results of the 5-CV experiment demonstrate the excellent performance of our proposed model in disease-lncRNA interaction prediction tasks.

**Table 1:** Results of NAGTLDA 5-CV

Fold	AUC	AUPR	F1-score	ACC	Recall	Spec.	Prec.
1	0.9523	0.9543	0.8806	0.8803	0.8831	0.8775	0.8782
2	0.9556	0.9559	0.8834	0.8756	0.9424	0.8089	0.8314
3	0.9538	0.9547	0.8855	0.8794	0.9332	0.8256	0.8425
4	0.9526	0.9523	0.8784	0.8756	0.8979	0.8534	0.8596
5	0.9509	0.9510	0.8823	0.8817	0.8872	0.8761	0.8775
Average	0.9531	0.9537	0.8820	0.8785	0.9088	0.8483	0.8578

Several hyperparameters are included in the model, including the final embedding dimension (*dim*), maximum smoothing steps (*k*), learning rate (*lr*), encoding dimension for SDNE (*nhid*), number of Transformer layers (*L*1 and *L*2), number of attention heads for multi-head attention (*Head*1 and *Head*2), *r*-value for NAFS, and weight decay for the optimizer. The best settings of hyperparameter optimization are presented in Table 2. The optimal parameter values are bolded, and these optimal parameters were chosen based on the model AUC.

**Table 2:** Hyperparameter setting of NAGTLDA

	Hyperparameter	Setting
NAFS	Threshold of lncRNA network *ɑ*	[0.3, 0.4, 0.5, **0.6**, 0.7, 0.8, 0.9]
	Threshold of disease network *β*	[0.3, 0.4, 0.5, **0.6**, 0.7, 0.8, 0.9]
	Maximum smoothing steps *k*	[2, 3, 4, 5, 6, **7**, 8, 9]
	List of *r* value	[**{0,0.1,0.2,0.3,0.4,0.5}**, {0.3,0.4,0.5}]
SDNE	First-order loss parameter *alpha*	1e-6
	Coding dimension *nhid1*	[32, **64**, 128, 256]
	Regularization term parameters *nu1*	1e-5
	Regularization term parameters *nu2*	1e-4
NAGTLDA	Learning rate *lr*	0.001
	Random *seed*	50
	Dropout	0.4
	Adam optimizer *weight-decay*	5e-3
	Number of layers of global-level embedding *L1*	[1, 5, **10**, 15]
	Number of layers of global-level embedding fusion *L2*	[15, 10, **20**, 25]
	Number of heads of global-level embedding *H1*	[4, **8**, 16, 32]
	Number of heads of global-level embedding fusion *H2*	[16, 32, **64**, 128]
	Feature embedding size *out-dim*	[32, **64,** 128**,** 256, 512]
	Epoch	150

### Parameter analysis

**Fig. 3:**
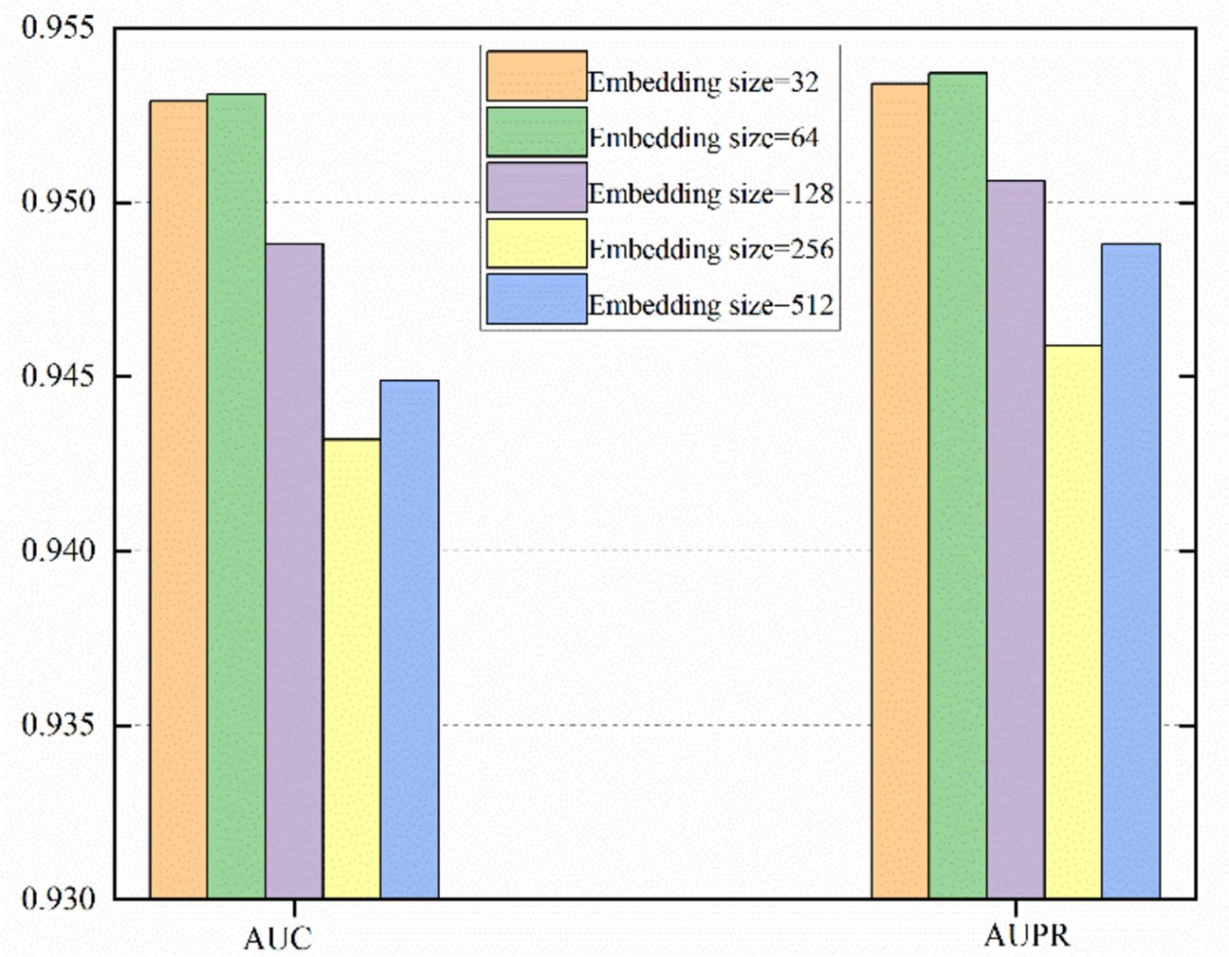
The effect of different embedding dimensions on the AUC and AUPR of NAGTLDA

During the process of setting hyperparameters, we found that certain parameter values have a noticeable impact on the model performance. For instance, we analyzed the dimensions of the final node features, as shown in Fig. 3. We compared different dimension values ($dim\in \{32, 64 ,128, 256, 512\}$) and found that when *dim* = 64, the AUC and AUPR values are highest. Selecting an appropriate dimension to represent node features is crucial. If the dimension is too small, the distinguishability between nodes may not be clear. However, if the dimension is too large, it can result in a significant amount of redundant information. Therefore, the choice of embedding dimension as a hyperparameter is also vital for the model.

**Fig. 4:**
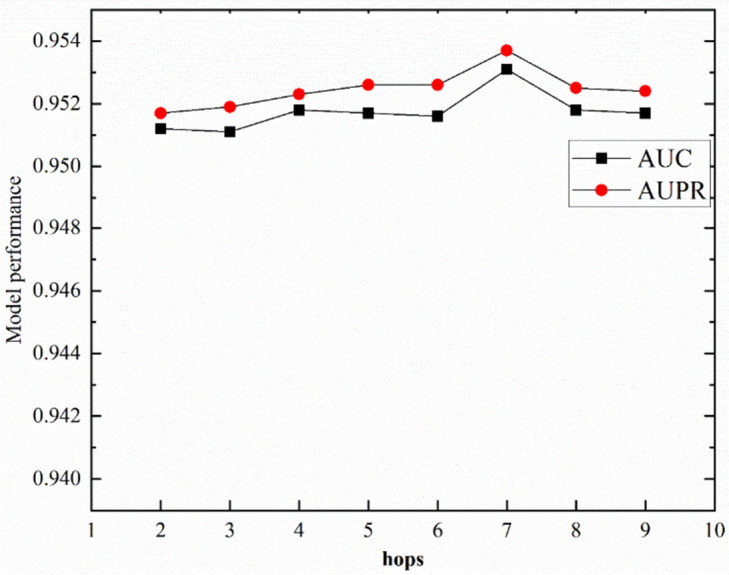
The effect of different maximal smoothing steps on the AUC and AUPR of NAGTLDA

Then, we analyzed the maximum number of smoothing steps in NAFS, as shown in Fig. 4. The maximum number of smoothing steps indicates the number of neighbours aggregated in the process of aggregating neighbour nodes, which is equivalent to aggregating multi-order neighbours. We found that when *hops* = 7, the values of AUC and AUPR are the highest. When hops are greater than 7, they show a decreasing trend, and when they are less than 7, they show an increasing trend. After each smoothing, the following node features will contain all the previous smoothing information, so the number of smoothing steps is also very important for the learning of feature embedding.

**Fig. 5:**
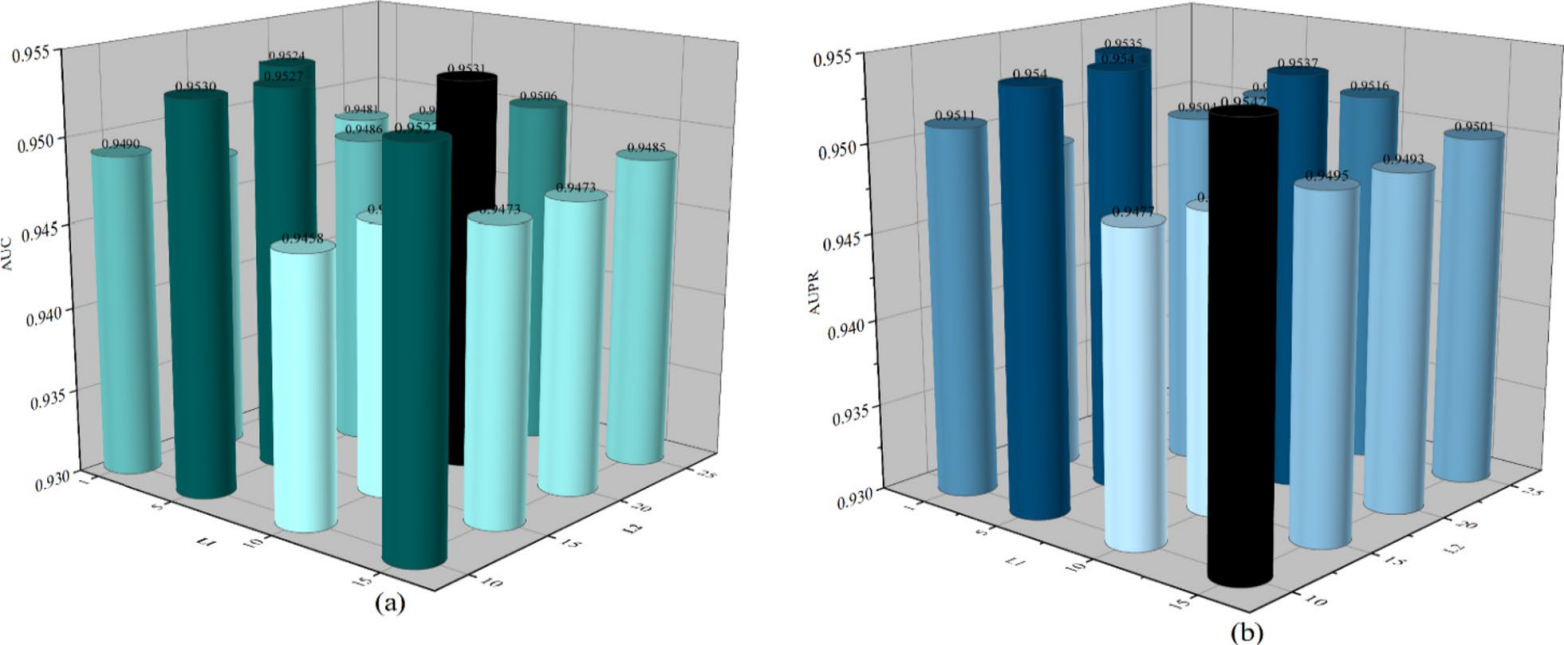
NAGTLDA performance under various Transformer layers

**Fig. 6:**
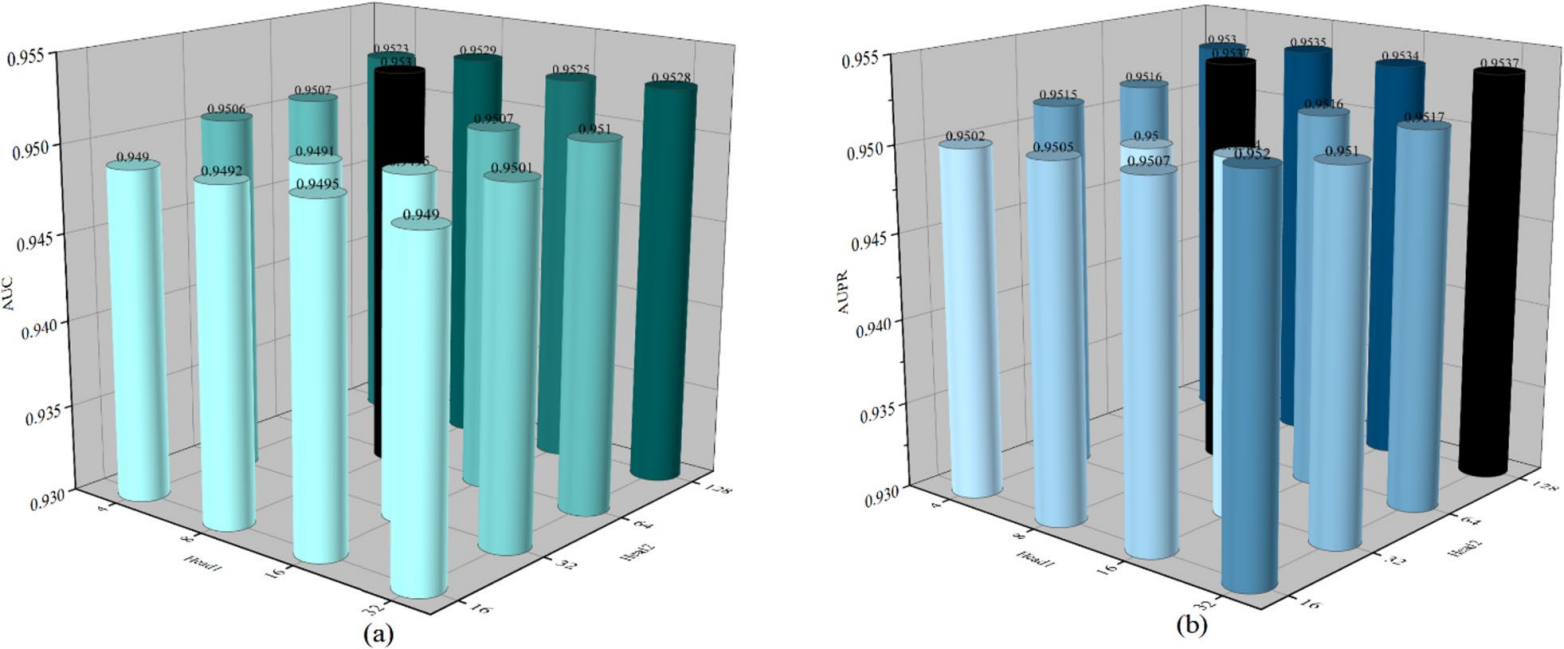
NAGTLDA performance under various heads of multi-head attention

In our model, we introduced the Transformer module, which includes a multi-head attention mechanism that provides us with a global perspective, enabling us to perform global-level embedding learning. We used two instances of the Transformer module in our model, and we found that different combinations of layer numbers (*L*1 and *L*2) have a significant impact on the model’s performance. As shown in Fig. 5a, different layer numbers affect the model’s AUC, while Fig. 5b illustrates the impact of different values of *L*1 and *L*2 on AUPR. The highest AUC value is achieved when the combination of (*L*1, *L*2) is set to (10, 20), while the highest AUPR value is achieved when it is set to (15, 10). Additionally, different combinations of the quantity for the attention heads, *Head*1 and *Head*2, also affect the prediction efficiency of the model. As depicted in Fig. 6a, the varying combinations of *Head*1 and *Head*2 influence the AUC values, with the highest value observed when it is set to (8, 64). In Fig. 6b, we can observe that the highest AUPR value is achieved when the combination of *Head*1 and *Head*2 is (8, 64).

### Performance comparison with different ratios

**Fig. 7:**
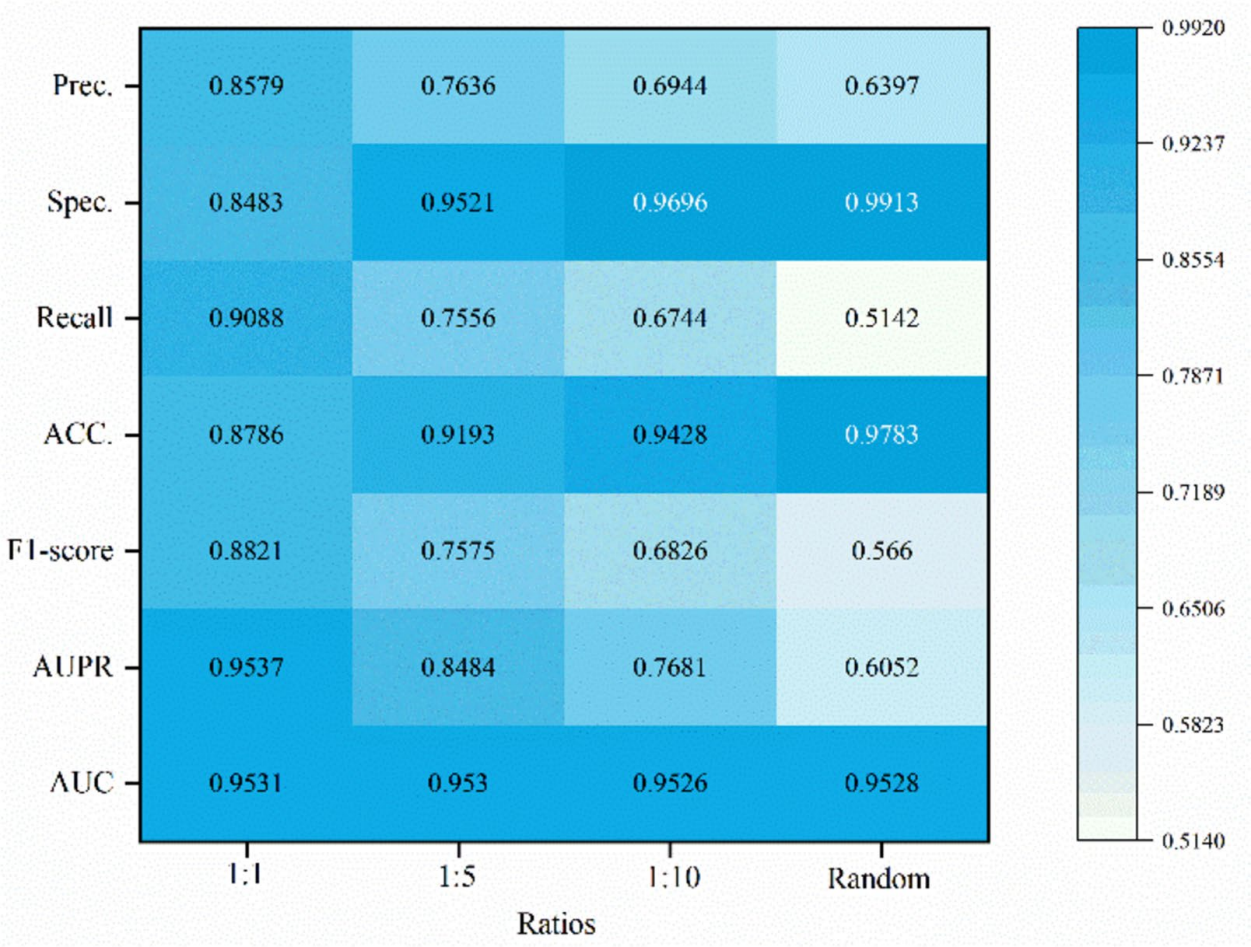
The effect of different ratios of positive and negative samples on the performance of NAGTLDA

The different proportions of negative and positive samples in each fold of cross-validation can also impact the model’s performance. Therefore, we set the proportions between positive samples and negative samples in each fold as follows: positive samples: negative samples = {1:1, 1:5, 1:10, *random*}, for experimental purposes. The detailed outcomes of the studies are presented in Fig. 7. We can observe that when the *ratio* = 1:1, indicating a balanced ratio of positive and negative samples, the AUC and AUPR values are the highest at 0.9531 and 0.9537, respectively, but the corresponding accuracy is the lowest. When the *ratio* = 1:5, the AUC and AUPR values are slightly lower than those of the *ratio* = 1:1, but the accuracy is slightly higher. When the *ratio* = 1:10, the AUC value is the lowest, but the accuracy is higher than the previous ratios. When the *ratio* is set to random, the AUC value is ranked third, and the AUPR value is the lowest, but the accuracy is the highest at 0.9783.

We speculate that the reason for these results may be due to the low proportion of positive samples in the experimental dataset. If we balance the positive and negative samples in each fold, it leads to the smallest quantity of training data in each fold, resulting in the lowest model accuracy. As the proportions between positive and negative samples decrease, the quantity of training data in each fold also decreases, leading to a decrease in accuracy.

### Performance comparison with other methods

In our experiments, we compared our model with six state-of-the-art computational methods on a benchmark dataset D1 using a 5-CV approach, which are as follows:

- HGATLDA (2022) [55]: A meta-path-based heterogeneous graph attention network framework was used to perform interaction prediction between diseases and lncRNAs by constructing disease, lncRNA, and gene heterogeneity networks.
- SFGAE (2022) [56]: A graph self-encoder was utilized for feature learning of nodes and self-featured representations of miRNAs and diseases were constructed for association prediction between miRNAs and diseases.
- VGAELDA (2021) [57]: An end-to-end computational model based on a variational self-encoder and graph self-encoder was adopted to predict the relationships between diseases and lncRNAs.
- LAGCN (2020) [58]: A layer-attentive graph convolution network was used to synthesize multisource similarity to construct heterogeneous network for association prediction between drugs and diseases.
- LDA-LNSUBRW (2020) [59]: A computational method based on unbalanced double random wandering and linear neighborhood similarity for association prediction between diseases and lncRNAs.
- CNNLDA (2019) [29]: A dual convolutional neural network model based on an attention mechanism that integrates multiple sources of data was used to excavate the associations between diseases and lncRNAs.

For benchmark dataset, the D1 downloaded from the Lnc2Cancer [16], LncRNADisease [17] and GeneRIF [47]. The dataset utilized in this study was sourced from the previous research conducted by Fu et al. [46] on lncRNA-disease association prediction. The dataset comprises 240 lncRNAs, 412 diseases, and 2,697 experimentally validated lncRNA-disease interactions. The semantic similarity data for all diseases is obtained from MeSH.

**Fig. 8:**
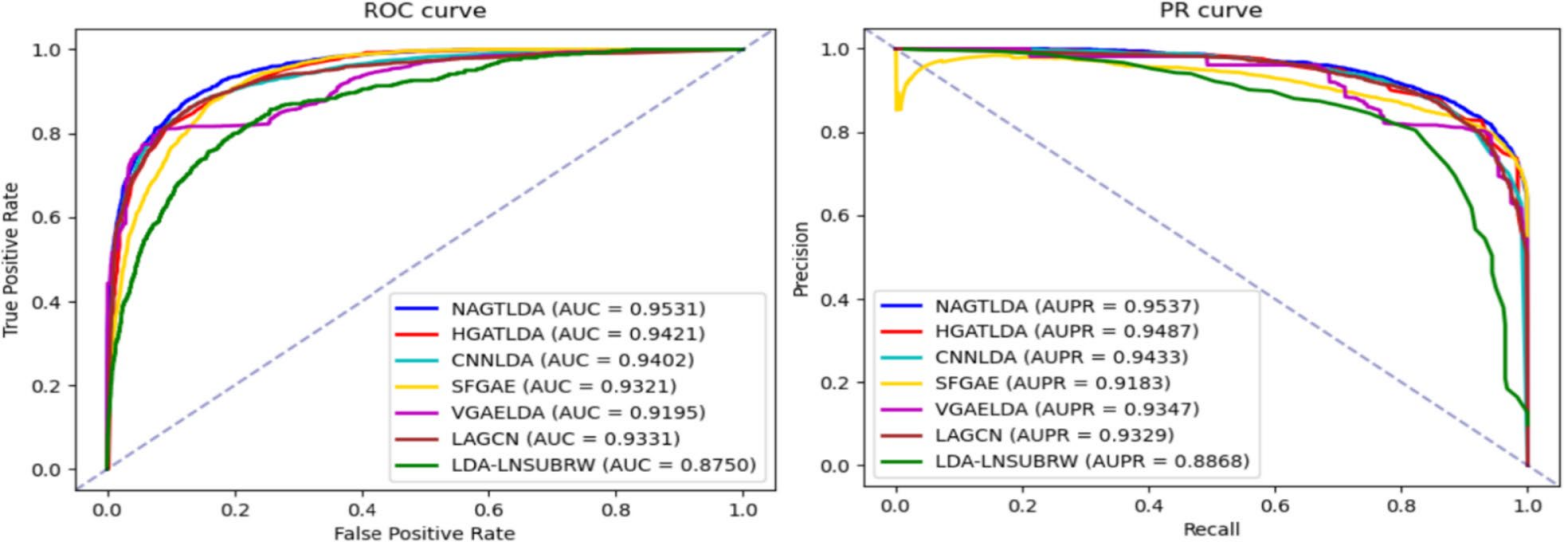
ROC curve and PR curve of the proposed method and six baselines under the 5-CV settings

**Fig. 9:**
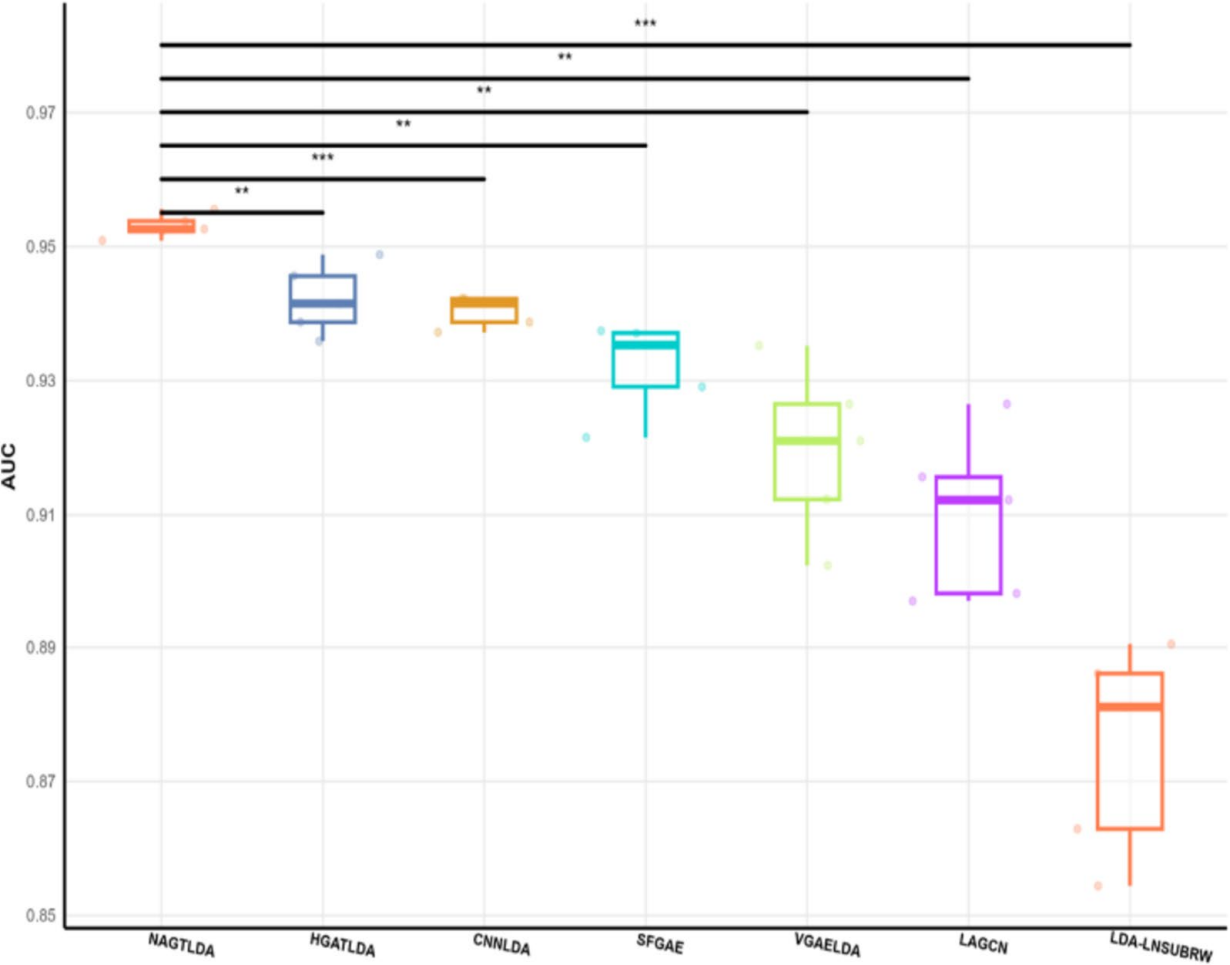
Significance analysis of other models with NAGTLDA on the D1 dataset

In the benchmark dataset D1 experiments, we compared different models using two evaluation metrics, namely, AUC and AUPR, to facilitate better comparison between models. The experimental results are presented in Table 3, where we highlight the highest results. It can be observed that our proposed NAGTLDA model achieves the highest AUC and AUPR values. This improvement can be attributed to the utilization of a Transformer for global learning during the process of learning node features. NAGTLDA outperforms LDA-LNSUBRW by 8.92% in AUC and 5.51% in AUPR. Figure 8 shows the AUC and AUPR curves of all models obtained through 5-CV experiments. It is evident from the figure that NAGTLDA outperforms other models in terms of performance. To visually highlight the performance disparity between NAGTLDA and existing state-of-the-art methods, we conducted a significance analysis of their AUC values, represented in Fig. 9 (* denotes *P* < 0.05, ** denotes *P* < 0.01, *** denotes *P* < 0.001). Notably, the significance levels of NAGTLDA compared to other methods are consistently high, ranging from a minimum significance of *P* < 0.05 to a maximum significance of *P* < 0.001. The improvement in the performance of our model has a significant enhancement for uncovering unknown lncRNA-disease associations. Hence, we can infer that our proposed model demonstrates excellent performance and serves as an effective computational approach for predicting disease-lncRNA associations.

**Table 3:** Performance comparison between our proposed method and six baselines under 5-CV settings

Models	AUC	AUPR
NAGTLDA	**0.9531**	**0.9537**
HGATLDA	0.9421	0.9487
CNNLDA	0.9402	0.9433
SFGAE	0.9321	0.9183
VGAELDA	0.9195	0.9347
LAGCN	0.9099	0.8891
LDA-LNSUBRW	0.8750	0.8868

Compared with these state-of-the-art methods, our model exhibits a significant performance advantage, as confirmed in the experiments above. The enhancement in performance can be attributed to the following unique contributions: NAFS is utilized to learn local features of nodes, simplifying the model training process and enhancing effectiveness. Moreover, the incorporation of network structure encoding enhances the efficiency of graph node information learning. Lastly, the application of the Transformer architecture allows for the learning of global information of nodes in the graph. The global and local features are then adaptively and efficiently fused using a multi-head attention approach, resulting in comprehensive feature information for diseases and lncRNAs.

### Performance on other datasets

To further validate the performance and generalization ability of the NAGTLDA model, we performed experiments on a larger lncRNA-disease association dataset D2 and a miRNA-disease association dataset D3, as shown in Table 4.

- D2: We screened the data from the databases of known lncRNA-disease associations, including LncRNADisease v2.0 [60] and Lnc2Cancer v3.0 [61], known lncRNA-miRNA associations from Encori [62] and NPInter V4.0 [63], and known miRNA-disease associations from HMDD v3.2 [64]. All disease names were converted to standard MeSH disease terms to facilitate the calculation of semantic similarity between the diseases. After removing redundant data, the final merger yielded 861 lncRNAs, 432 diseases, and 4516 known lncRNA-disease associations. The features used to make semantic similarity of diseases in the model are obtained from MeSH.
- D3: The known miRNA-disease association data were downloaded from the HMDD v3.2 database [64], and we obtained 788 miRNAs, 374 diseases, and 8968 corresponding known associations from the screening. The features used to make semantic similarity of diseases in the model are obtained from MeSH.

**Table 4:** Details about datasets

Datasets	ncRNA Types	ncRNAs	Diseases	Associations	Sparsity
D1	lncRNA	240	412	2697	2.728%
D2	lncRNA	861	432	4516	1.214%
D3	miRNA	788	374	8968	3.043%

We conducted 5-fold cross-validation experiments on the D2 and D3 datasets, and the results are presented in Table 5. Comparing the experimental outcomes of the original dataset with the D2 dataset, we observed that the model performs better on D2. This improved performance can be attributed to the incorporation of the Transformer structure into the NAGTLDA model, enhancing its performance on larger datasets. The Transformer, originally designed for large-scale natural language processing tasks, brings notable advantages to our model, allowing it to excel on larger datasets.

**Table 5:** NAGTLDA performance under D1 and D2 datasets

Datasets	AUC	AUPR	F1-score	ACC	Recall	Spec.	Prec.
D2	0.9630	0.9624	0.9177	0.9170	0.9258	0.9083	0.9103
D3	0.9419	0.9437	0.8746	0.8724	0.8899	0.8548	0.8601

On the D3 dataset, we achieved remarkable results with AUC and AUPR values exceeding 0.94, while the F1-score reached 0.8746. These outcomes indicate that our model possesses strong generalization capabilities. It not only performs well in predicting lncRNA-disease associations, which is the primary focus of our study, but also demonstrates high performance on other non-coding RNA datasets.

We established independent validation sets to assess the performance of our model, following the methodology outlined by Fu et al. [65]. For the D1 dataset, which contains 2697 positive samples, we initially selected 20% of the positive samples and the same number of negative samples to construct an independent balanced validation set (B-validation set). The remaining samples were utilized for training. Subsequently, we randomly extracted 20% samples from the D1 dataset to create an unbalanced independent validation set (Unb-validation set), while the remaining samples served as the training set. The experimental results on these two independent validation sets are summarized in Table 6. We assessed the model’s performance on the two independent validation sets in comparison to its performance on the benchmark dataset. Notably, there was a decrease in performance on the independent validation sets, specifically in terms of the two primary metrics, AUC and AUPR. Despite this decrease, the model still demonstrated relatively good results. Furthermore, the AUC and AUPR on the unbalanced independent validation set were slightly lower than those on the balanced validation set. This trend was observed in both balanced and unbalanced datasets, suggesting the need to explore strategies for choosing an optimal ratio of positive and negative samples to enhance the comprehensiveness of model comprehensiveness during training.

**Table 6:** Performance of NAGTLDA on D1 dataset and independent validation set

Datasets	AUC	AUPR	F1-score	ACC	Recall	Spec.	Prec.
D1	0.9531	0.9537	0.8820	0.8785	0.9088	0.8483	0.8578
B-validation set	0.9509	0.9510	0.8823	0.8817	0.8872	0.8761	0.8775
Unb-validation set	0.9505	0.5839	0.5437	0.9763	0.5250	0.9889	0.5717

**Fig. 10:**
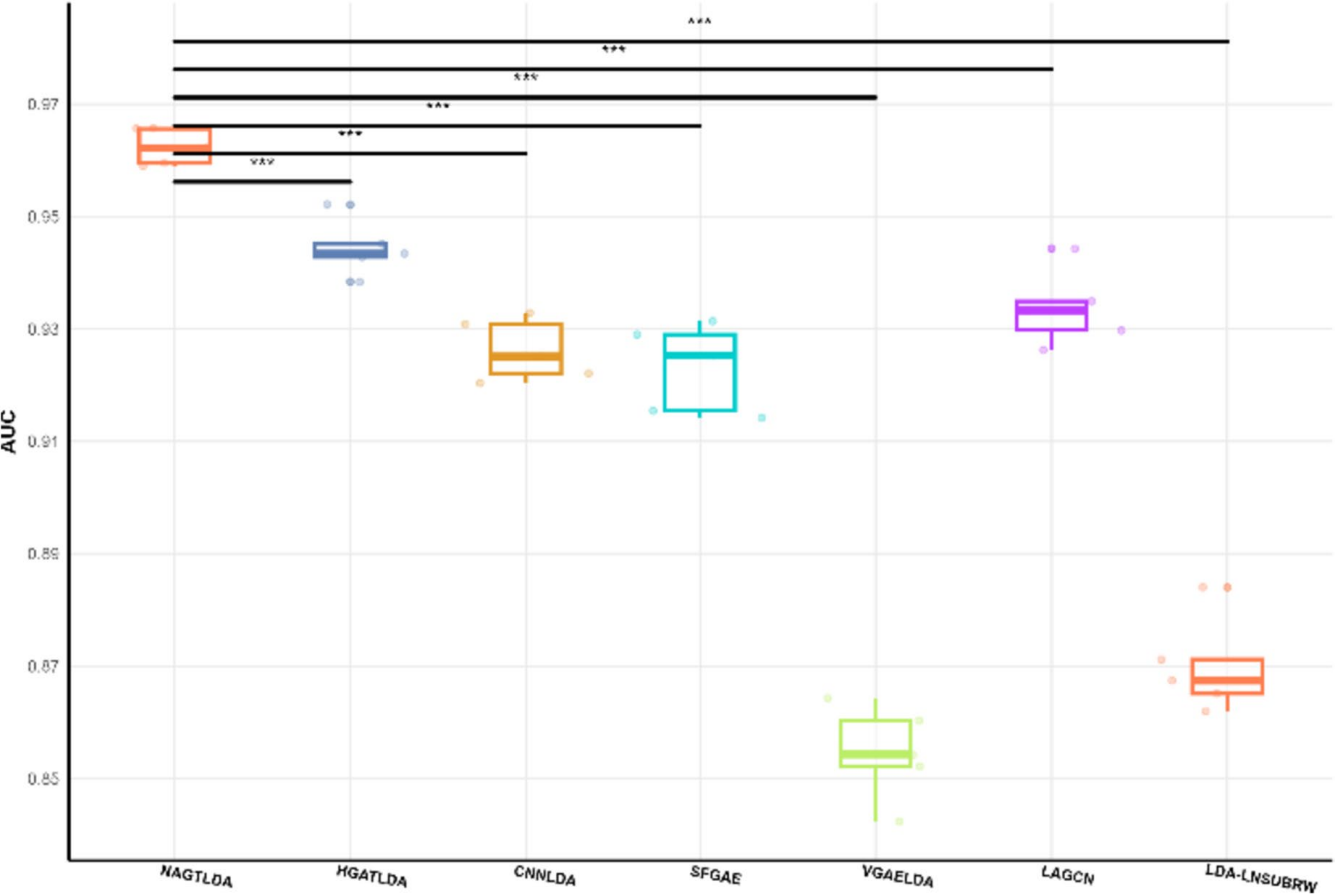
Significance analysis of other models with NAGTLDA on the D2 dataset

**Fig. 11:**
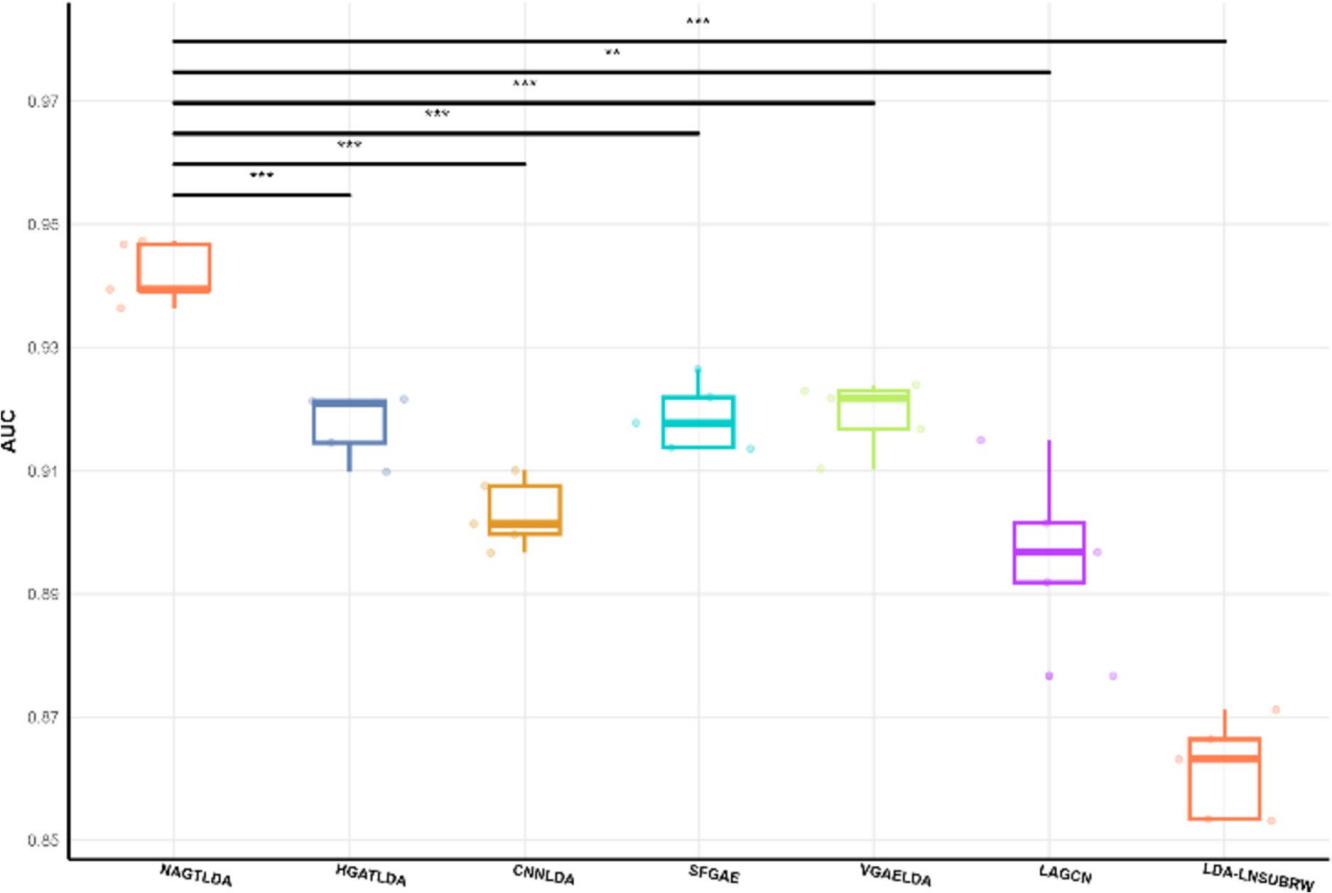
Significance analysis of other models with NAGTLDA on the D3 dataset

After comparing NAGTLDA with other state-of-the-art models in previous experiments on the D1 dataset, we extended our evaluation to two larger datasets, D2 and D3. We analyzed the significance of their AUC values, as illustrated in Figs. 10 and 11, to assess computational efficiency and scalability across models. Notably, NAGTLDA exhibited remarkable significance compared to other models on both datasets, with particularly noteworthy results on the D2 dataset, where the significance compared to other state-of-the-art models reached *P* < 0.001.

The reason for the strong scalability of our model is as follows: (1) Our model applied SDNE to learn the structure coding based on the specific network. (2) We leveraged the graph transformer structure to learn global level features, which can adaptively learn the features of nodes and has a very powerful learning capability. (3) We added NAFS to learn local features to make the model more scalable by flexibly learning the information of different nodes.

However, there are some limitations of our proposed model on large dataset. Large datasets are commonly imbalanced in positive and negative samples, which requires to introduce multi-source features to compensate for the shortcomings of sparse positive samples. Moreover, there are many hyperparameters in the model, and the model application on large datasets may cause overfitting phenomenon for too many parameters.

### Feature visualization

**Fig. 12:**
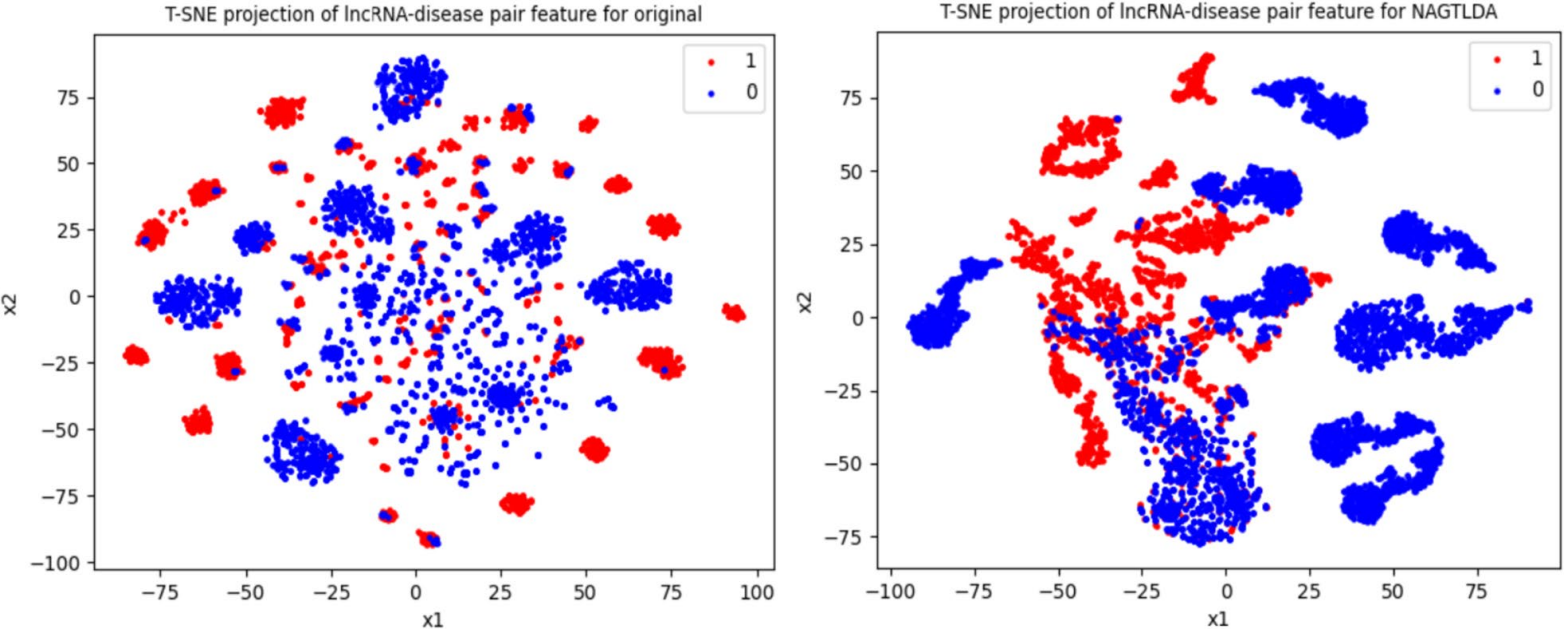
Comparison of visualization features of lncRNA-disease pairs obtained by NAGTLDA and the original

To display the effectiveness of our proposed model more specifically and graphically, we visualize the lncRNA-disease pair features learned by the model for comparison. We used t-SNE [66] to downscale the lncRNA-disease pair features and plot them in the two-dimensional plane to compare the learned pair features with the original pair features. As shown in Fig. 12, we visualize the original pair features (left) and the learned pair features (right). In the visualization, we distinguish the negative samples from the positive samples with different color dots, and we can observe that the lncRNA-disease pairs learned by NAGTLDA are more concentrated and distinguishable than the original positive and negative samples respectively. This also indicates that our model is meaningful and interpretable for disease and lncRNA feature learning.

### Ablation experiments

To assess the influence of each module on the model performance and its importance, three sets of ablation experiments were performed for validation.

The first set of ablation experiments is to remove a module from the initial model to construct a comparison model, and each new comparison model is described as follows:

- Remove T1: Remove the Transformer module that performs global-level embedding of heterogeneous networks.
- Remove lncRNA-NAFS: Remove the NAFS module that performs local-level embedding of the lncRNA-integrated similarity network.
- Remove disease-NAFS: Remove the NAFS module that performs local-level embedding of the disease-integrated similarity network.
- Remove lncRNA-SDNE: Remove the SDNE module that encodes the structure of the lncRNA-integrated similarity network.
- Remove disease-SDNE: Remove the SDNE module that encodes the disease-integrated similarity network structure.

**Fig. 13:**
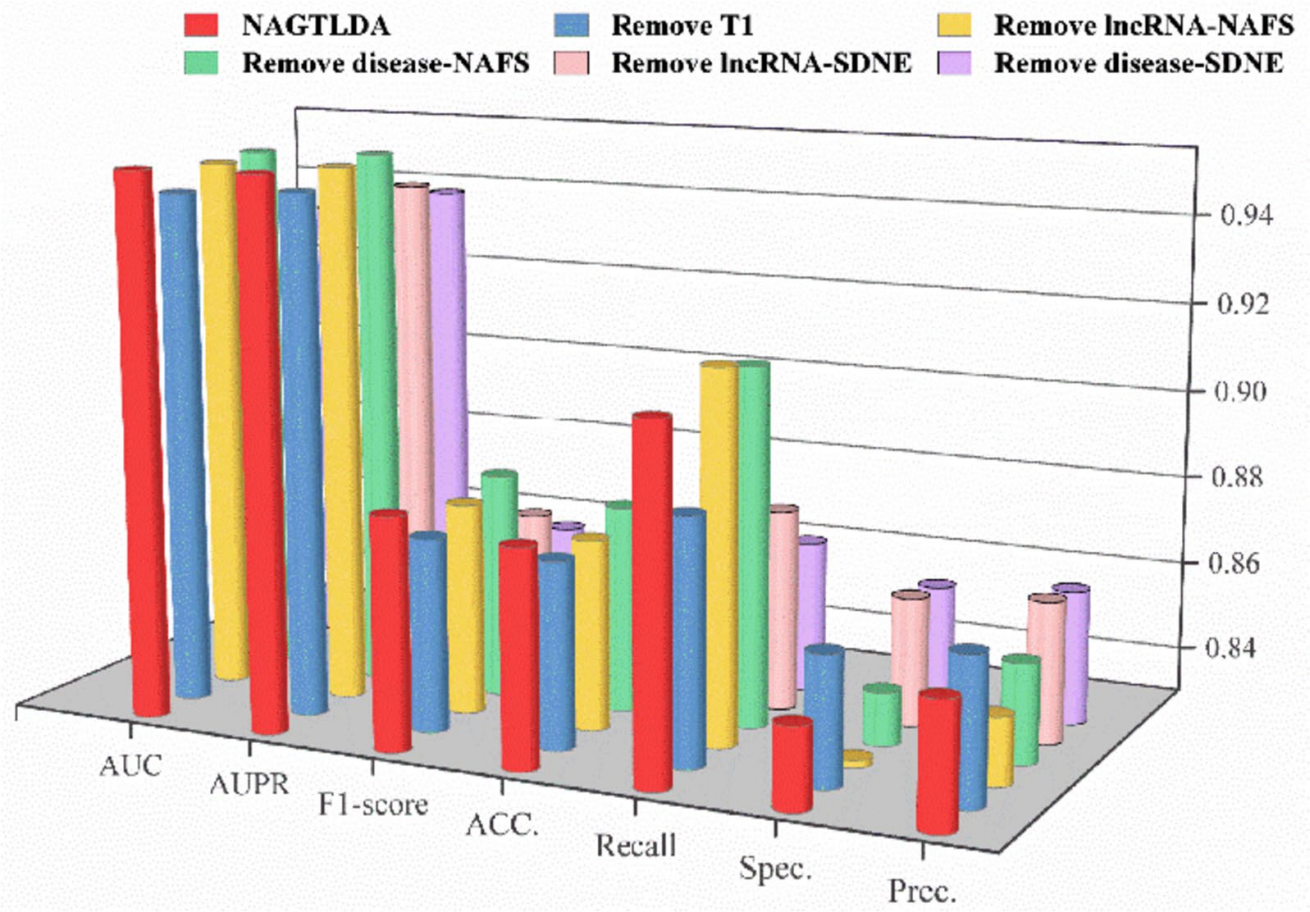
Comparison between NAGTLDA and multiple variant models

The results obtained from the experiments are presented in Fig. 13 and Table 7, and the original NAGTLDA model has excellent results compared to other comparable models. For example, on both the AUC and AUPR, NAGTLDA outperforms remove disease-SDNE by values of 0.0181 and 0.0133, respectively. We observe that encoding the network structure information exerts the most significant impact on the overall model performance. Consequently, the acquisition of node-level information within the network holds great importance. However, a comprehensive understanding of the network’s structural information also emerges as a vital component. The overall performance of the new model formed by removing a module is lower than that of the original model, thus proving the effectiveness of our use of Transformer layer for global-level embedding, NAFS for local-level embedding, and SNDE for network structure encoding.

**Table 7:** Performance between NAGTLDA and multiple variant models

Models	AUC	AUPR	F1-score	ACC	Recall	Spec.	Prec.
NAGTLDA	**0.9531**	**0.9537**	**0.8821**	**0.8786**	0.9088	0.8483	0.8579
Remove T1	0.9462	0.9479	0.8736	0.8720	0.8850	**0.8591**	**0.8628**
Remove lncRNA-NAFS	0.9510	0.9517	0.8777	0.8728	**0.9139**	0.8316	0.8451
Remove disease-NAFS	0.9520	0.9528	0.8809	0.8767	0.9113	0.8420	0.8523
Remove lncRNA-SDNE	0.9394	0.9438	0.8683	0.8672	0.8754	0.8590	0.8617
Remove disease-SDNE	0.9350	0.9404	0.8617	0.8611	0.8646	0.8576	0.8601

**Fig. 14:**
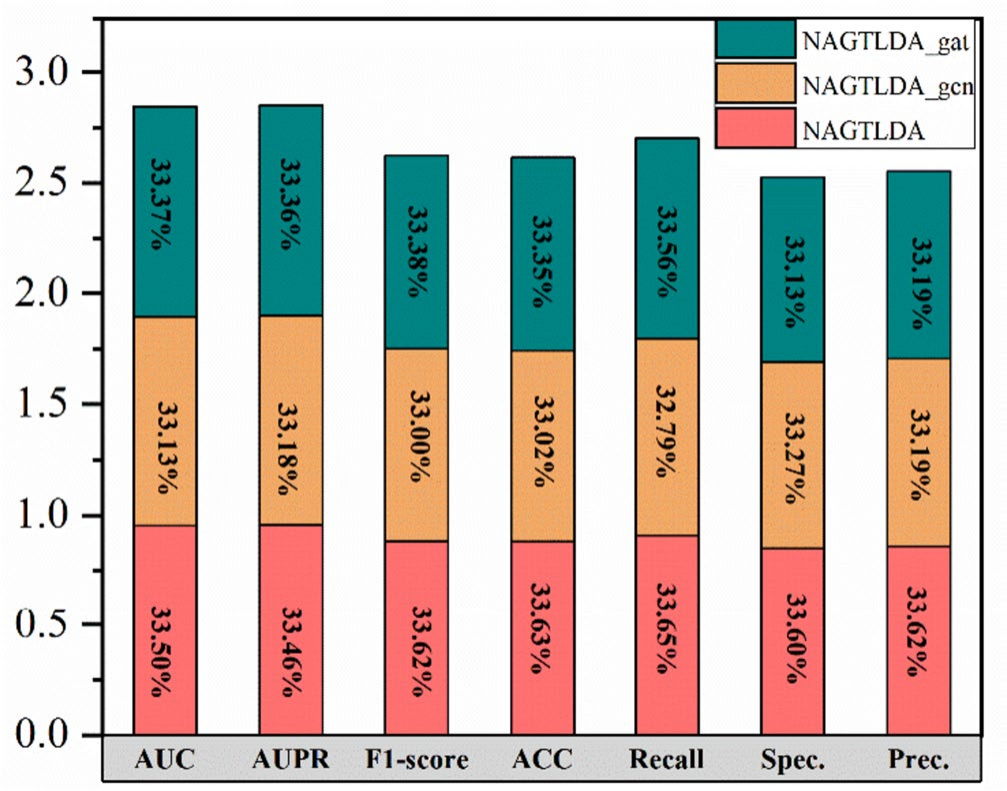
Comparison results of NAGTLDA, NAGTLDA_gcn and NAGTLDA_gat

The second set of ablation experiments was conducted by replacing the method used for local-level embedding in the model with the classical GCN and GAT in graph neural networks to construct the comparison models: NAGTLDA_gcn and NAGTLDA_gat. As shown in Table 8 and Fig. 14, NAGTLDA performs better than the variant model. Specifically, NAGTLDA is 0.0106 higher than NAGTLDA_gcn in terms of AUC value, 0.0079 higher than NAGTLDA_gat in terms of AUPR, and 0.0158 higher than NAGTLDA_gcn in accuracy. NAGTLDA compared to NAGTLDA_gcn and NAGTLDA_gat in F1- score is the highest, and the F1-score is a benchmark indicator for the comprehensive ability of the model, so the original model is a better choice. Combining the outcomes of the first set of ablation experiments and the present set of experiments, it can be concluded that using NAFS for embedding learning of node features is an efficient learning method, and it also proves the effectiveness and efficiency of using NAFS in the whole model.

**Table 8:** Performance of NAGTLDA based on different local-level embeddings methods

Models	AUC	AUPR	F1-score	ACC	Recall	Spec.	Prec.
NAGTLDA	**0.9531**	**0.9537**	**0.8821**	**0.8786**	**0.9088**	**0.8483**	**0.8579**
NAGTLDA_gcn	0.9425	0.9458	0.8658	0.8628	0.8857	0.8398	0.8470
NAGTLDA_gat	0.9493	0.9507	0.8757	0.8713	0.9065	0.8363	0.8470

**Fig. 15:**
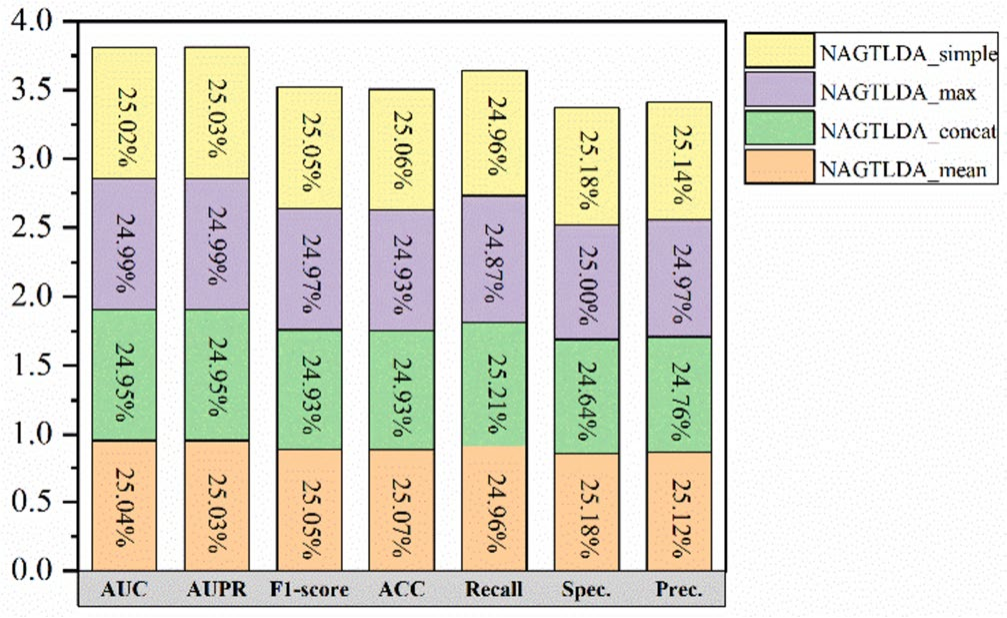
Comparison results of NAGTLDA, NAGTLDA_concat, NAGTLDA_max and NAGTLDA_simple

The third set of ablation experiments is conducted for NAFS. We input a set of r values to obtain a set of different node feature representations, and we can use different ways to process this set of node feature representations. NAGTLDA_concat, NAGTLDA_max and NAGTLDA_simple represent the use of concatenate, max and simple operations, respectively. The simple operation means inputting only one r value to one experimental result. The detailed experimental outcomes are presented in Fig. 15 and Table 9. Six of the seven evaluation metrics in the experimental results are the highest when the mean operation is used.

**Table 9:** Performance of NAFS based on different fusion methods

Models	AUC	AUPR	F1-score	ACC	Recall	Spec.	Prec.
NAGTLDA_mean	**0.9531**	**0.9537**	**0.8821**	**0.8786**	0.9088	**0.8483**	**0.8579**
NAGTLDA_concat	0.9498	0.9506	0.8778	0.8739	0.9054	0.8424	0.8521
NAGTLDA_max	0.9515	0.9521	0.8792	0.8739	**0.9177**	0.8301	0.8450
NAGTLDA_simple	0.9525	0.9535	**0.8821**	0.8785	0.9087	**0.8483**	0.8573

### Case study

In the previous sections, we tested and confirmed the effectiveness of NAGTLDA. Now, we evaluate NAGTLDA’s ability to excavate unknown relationships between diseases and lncRNAs. We chose four common diseases, which are prostate cancer, colon cancer, breast cancer, and colorectal cancer, as case studies from the dataset. We trained the model with 2797 observed lncRNA-disease relationships as instances for training and then made predictions for unknown potential associations. We extracted the top 15 candidate lncRNAs for each disease and validated the results using three benchmark databases: LncRNADisease v2.0 [60], Lnc2Cancer 3.0 [61], and MNDR v3.1 [67].

The exact cause of colon cancer is still unknown, but studies and research have shown that the risk of developing the disease increases with age, obesity, and cancer in other parts of the body. As research continued, researchers found that colon cancer is closely linked to several lncRNAs. For example, CYTOR and the corresponding protein binding can contribute to the metastasis of colon cancer [68], and HOXB-AS3 expression can inhibit the growth of colon cancer [69]. The experimental outcomes are presented in Table 10, where 14 of the top 15 candidate lncRNAs have been confirmed.

**Table 10:** The top 15 predicted lncRNAs associated with colon cancer

LncRNA name	Evidence	Rank	LncRNA name	Evidence	Rank
GAS5	PMID:28722800	1	DANCR	PMID:30127873, 32423468	9
PVT1	PMID:25043044, 29552759	2	KCNQ1OT1	PMID:31040703	10
UCA1	PMID:17416635, 26885155	3	HULC	PMID:27496341, 30551459	11
CDKN2B-AS1	PMID:23416462, 33529508	4	XIST	PMID:29679755	12
NEAT1	PMID:26164760, 31173354	5	AFAP1-AS1	PMID:30588252	13
TUG1	PMID:27634385, 31697952	6	BCYRN1	PMID:29625226	14
HOTTIP	Unknown	7	MIR155HG	PMID:27821766	15
MIR17HG	PMID:35249533	8			

The most prevalent malignancy is prostate cancer in the male urological system, which is highly prevalent in older men, but its etiology has not yet been fully identified. Researchers have found that prostate cancer is closely related to the expression of lncRNAs. For example, the expression of MAGI2-AS3 and MEG3 in lncRNAs inhibits the development of prostate cancer [70, 71], and MNX1-AS1 indirectly promotes the development of prostate cancer through expression [72]. We used it as the second disease in the case study, and the experimental outcomes are presented in Table 11. Thirteen of the top 15 candidate lncRNA species we identified have been confirmed by the relevant literature.

**Table 11:** The top 15 predicted lncRNAs associated with prostate cancer

LncRNA name	Evidence	Rank	LncRNA name	Evidence	Rank
MIR17HG	PMID:27556357	1	CCAT1	PMID:28945760, 29863242	9
XIST	PMID:16261845, 27507663	2	WT1-AS	Unknown	10
HCP5	PMID:31746434, 34285549	3	CCAT2	PMID:27558961, 28244168	11
BCYRN1	PMID:32705287	4	SOX2-OT	PMID:31623830, 32407168	12
GHET1	PMID:30609158	5	LINC00675	PMID:30963639	13
BANCR	Unknown	6	CASC2	PMID:29373811	14
AFAP1-AS1	PMID:31081081, 31669642	7	SPRY4-IT1	PMID:25307116, 26503110	15
TP53COR1	PMID:25999983, 27976428	8			

Breast cancer is the most common cancer among women. According to research, obesity, excessive alcohol consumption, and overnutrition all increase the incidence of breast cancer, but thus far, medical researchers have not found the exact cause of cancer. With the persistent expansion of bioclinical technology, growing number of lncRNAs related to breast cancer have been discovered. For example, the distant metastasis-free survival, overall survival, and progression-free survival of breast cancer patients are strongly associated with high expression of BCAR4, LUCAT1, and TINCR [73–75]. LINC00511 binds to the MMP13 protein to promote breast cancer cell migration and proliferation [76]. We used breast cancer as the third type of disease in the case study, and the experimental outcomes are presented in Table 12. All of top 15 candidate lncRNAs have been validated by the relevant literature.

**Table 12:** The top 15 predicted lncRNAs associated with breast cancer

LncRNA name	Evidence	Rank	LncRNA name	Evidence	Rank
TUG1	PMID:27791993, 27848085	1	MIR155HG	PMID:23246696, 32165090	9
HULC	PMID:27986124, 30957286	2	HNF1A-AS1	PMID:31837323, 32319789	10
MIR17HG	PMID:36943627	3	TP53COR1	PMID:22487937, 26656491	11
BANCR	PMID:29565494, 29805676	4	HCP5	PMID:32165090	12
IGF2-AS	PMID:31319040, 33175607	5	PCAT1	PMID:28989584, 31319040	13
DANCR	PMID:27716745, 28978036	6	GHET1	PMID:29843220, 30787968	14
WT1-AS	PMID:18708366	7	CASC2	PMID:29523222, 30106139	15
NPTN-IT1	PMID:30280783	8			

Colorectal cancer is the third most common malignancy in the world, and its incidence is relatively similar in men and women. The majority of the population suffers from the disease due to lifestyle habits, and a very small percentage is due to genetic factors. Colorectal cancer ranks second in the number of deaths caused by malignant tumors. Researchers have found through numerous clinical trials that ITGB8-AS1 combined with the corresponding signals can contribute to the growth and metastasis of colorectal cancer [77] and that GAS5 and YAP phosphorylation and degradation interact to inhibit the development of colorectal cancer [78]. We used it as the fourth disease in our case study, and the experimental outcomes are presented in Table 13, where 13 of the top 15 candidate lncRNAs we selected have been validated by the relevant literature.

**Table 13:** The top 15 predicted lncRNAs associated with colorectal cancer

LncRNA name	Evidence	Rank	LncRNA name	Evidence	Rank
SPRY4-IT1	PMID:27391336, 27621655	1	LINC00687	Unknown	9
MIR17HG	PMID:31409641	2	IGF2-AS	PMID:32853944	10
CDKN2B-AS1	PMID:26708220, 27286457	3	TRERNA1	PMID:31933996, 33833618	11
ZEB1-AS1	PMID:28618933, 28967064	4	MIR194-2HG	Unknown	12
PANDAR	PMID:27629879, 28106228	5	LINC00974	PMID:35907803	13
HNF1A-AS1	PMID:28791380, 29145164	6	CYTOR	PMID:27633443, 28078002	14
WT1-AS	PMID:30714675	7	PCAT1	PMID:23640607, 27591862	15
DBH-AS1	PMID:33549042	8			

## Discussion

In the present paper, we designed a NAGTLDA computational model to make inferences about unknown interactions between lncRNAs and diseases. Based on the experimental results, our model demonstrates promising performance, particularly in handling large datasets. The high scalability across varying sizes of datasets can be ascribed to the utilization of the graph Transformer architecture for extracting feature representations. This architecture possesses a highly expressive and adaptive learning capability, enabling it to learn diverse networks effectively.

However, our proposed model and the current study have some limitations. The limitations of our model are as follows: (1) The main framework of our model is built upon the Transformer architecture, requiring considerable computational power during the training process, particularly in practical applications involving large datasets. (2) The existence of numerous hyperparameters necessitates meticulous optimization and tuning, thereby augmenting the complexity of the training process. (3) Our model also relies on the initial similarity features of the nodes, which are calculated based on the association matrix. There are some limitations in the present field of lncRNA-disease association prediction as follows:(1) There are no true negative samples in the experimental data, and all the biological data are looking for true positive samples and not paying much attention to negative samples. Negative samples may be correct or they may be undetected false negatives. (2) The experimental results of computational modeling do not correlate very well with biological experiments, and better integration of computational modeling and biological experiments makes the results better interpretable. In future research, we can start by studying the dataset and exploring how to better represent the correlations between entities, which will result in a more accurate discovery of unknown associations. In addition, as medical science and technology continue to advance, the discovery of more unknown lncRNAs, represented as isolated nodes, is anticipated. Moving forward, there is a pressing need to develop more comprehensive models that can accurately predict the associations between these isolated nodes and experimentally verified disease nodes.

## Conclusions

In the model, we first framed a heterogeneous network consisting of diseases and lncRNAs, an integrated similarity network for diseases and an integrated similarity network for lncRNAs, and used NAFS to perform node-level embedding for each of the three networks. We also adopted SDNE to encode the structural information of the networks with the goal of utilizing the constructed networks more effectively. We then introduce the Transformer module for global-level embedding to explore potential unknown associations in the dataset and utilize the Transformer fusion mechanism with two levels of attention to perform global-level embedding fusion on the learned embeddings and network topology. We performed embedding learning on the network information from both local and global perspectives so that some potential associations can be better identified. Finally, a bilinear decoder is employed to fuse the node embedding representations of diseases and lncRNAs as input for lncRNA and disease association prediction. We also conducted experiments on the performance of our model, and the outcomes of the 5-CV and contrast to other baseline models confirm the excellent performance of our model. In the case study, NAGTLDA successfully predicted associations, such as NEAT1-colon cancer, SOX2-OT-prostate cancer, and WT1-AS-colorectal cancer, which were previously unknown in the dataset. He et al. [79] investigated the function of NEAT1 in colon cancer, and found that the expression of NEAT1 was significantly elevated in colon cancer cells in their experiments, which proved that NEAT1 indirectly promotes the occurrence of colon cancer. Song et al. [80] demonstrated that SOX2-OT inhibits the proliferation and metastasis of prostate cancer cells by interacting with other non-coding RNAs. This discovery provides a new therapeutic approach for the treatment of prostate cancer. Zhang et al. [81] experimentally demonstrated experimentally that WT1-AS was closely associated with overall survival in colorectal cancer. The correlation between WT1-AS and colorectal cancer was demonstrated on clinicopathological features and data modeling analysis, and WT1-AS can be used as a biomarker and therapeutic target for colorectal cancer prognosis. This proves that our proposed model performs very well in finding new therapeutic strategies for diseases and provides a solid foundation for biological experiments and clinical practice.

## Data Availability

For lncRNA-disease, the D1 dataset downloaded from the Lnc2Cancer [13]: http://www.bio-bigdata.net/lnc2cancer, LncRNADisease [14]: http://cmbi.bjmu.edu.cn/lncrnadisease and GeneRIF [38]: https://ftp.ncbi.nlm.nih.gov/gene/GeneRIF/, the D2 dataset screened from the databases of known lncRNA-disease associations, including LncRNADisease v2.0 [51]: http://www.rnanut.net/lncrnadisease, and Lnc2Cancer v3.0 [52]: http://www.bio-bigdata.net/lnc2cancer, known lncRNA-miRNA associations from Encori [53]: http://starbase.sysu.edu.cn/. and NPInter V4.0 [54]: http://bigdata.ibp.ac.cn/npinter, and known miRNA-disease associations from HMDD v3.2 [55]: http://cuilab.cn/hmdd. The miRNA-disease associations D3 are downloaded from the HMDD v3.2 database [55]: http://cuilab.cn/hmdd. The semantic similarity data for all diseases is obtained from MeSH at http://www.nlm.nih.gov. The code of NAGTLDA is provided on GitHub (https://github.com/ghli16/NAGTLDA).

## Abbreviations

lncRNA: Long non-coding RNA
NAFS: Node-adaptive feature smoothing
SDNE: Structural Deep Network Embedding
GCN: Graph Convolutional Network
DAG: Directed acyclic graph
AUPR: Area under precision-recall curve
AUC: Area under the receiver operating characteristic curve
5-CV: 5-Fold cross validation
DO: Disease Ontology
CNN: Convolution Neural Network
